# AGO2 localizes to cytokinetic protrusions in a p38-dependent manner and is needed for accurate cell division

**DOI:** 10.1038/s42003-021-02130-0

**Published:** 2021-06-11

**Authors:** Vasiliki I. Pantazopoulou, Anastasios D. Delis, Stella Georgiou, Stamatis N. Pagakis, Vicky Filippa, Eleni Dragona, Ismini Kloukina, Elias Chatzitheodoridis, Jonel Trebicka, Athanassios D. Velentzas, Maja Thiele, Sarantis Gagos, Dimitris Thanos, Sofia Tseleni-Balafouta, Dimitrios J. Stravopodis, Ema Anastasiadou

**Affiliations:** 1 Biomedical Research Foundation of the Academy of Athens, Athens, Greece; 2 National Technical University of Athens, Athens, Greece; 3 Goethe University Clinic, Frankfurt, Germany; 4 National and Kapodistrian University of Athens, Athens, Greece; 5 University of Southern Denmark, Odense, Denmark

**Keywords:** Cell division, RNAi

## Abstract

Argonaute 2 (AGO2) is an indispensable component of the RNA-induced silencing complex, operating at the translational or posttranscriptional level. It is compartmentalized into structures such as GW- and P-bodies, stress granules and adherens junctions as well as the midbody. Here we show using immunofluorescence, image and bioinformatic analysis and cytogenetics that AGO2 also resides in membrane protrusions such as open- and close-ended tubes. The latter are cytokinetic bridges where AGO2 colocalizes at the midbody arms with cytoskeletal components such as α-Τubulin and Aurora B, and various kinases. AGO2, phosphorylated on serine 387, is located together with Dicer at the midbody ring in a manner dependent on p38 MAPK activity. We further show that AGO2 is stress sensitive and important to ensure the proper chromosome segregation and cytokinetic fidelity. We suggest that AGO2 is part of a regulatory mechanism triggered by cytokinetic stress to generate the appropriate micro-environment for local transcript homeostasis.

## Abstract (web summary)

Pantazopoulou et al. find that AGO2 resides in open-ended tunneling nanotubes and close-ended cytokinetic bridges. At the latter location, AGO2 colocalizes with cell division components and the authors show that AGO2 depletion impairs cell division fidelity.

## Introduction

Argonaute (AGO) proteins are at the hub of RNA-induced silencing complex (RISC), a major contributor to the fine-tuning of gene expression due to posttranscriptional regulation^1^. AGOs are ubiquitously expressed and present strong evolutionary conservation. The human AGO family includes four different proteins in humans: AGO1, AGO2, AGO3, and AGO4, with extremely high homology (exceeds the 80% value over the entire protein-length) among all members. Although they share the same signature domains N, MID, PAZ, and PIWI, only AGO2 has proved to exert slicer-endonuclease and microRNA (miRNA) stabilization activities in humans, till recently. The epigenetic process is directed by small RNAs^2^, the anchoring of which into concrete binding pockets allows the AGO2 guidance toward the respective targets^3^. Higher eukaryotes feature a large diversity of AGOs and small RNAs, including miRNAs, which are implicated in a plethora of cellular processes, such as cell differentiation, transposon silencing, embryonic development, and cancer^1,3,4^. In the canonical pathway, the mature miRNAs are loaded onto AGO proteins in an ATP-dependent manner^5^. The miRNA active guide strand is considered to have the lower 5’ stability and interacts with AGO2 complex while the unloaded passenger strand is unwound from the guide strand, according to the degree of complementarity^6,7^. Dynamic alterations in mRNA structure, mRNA-folding, and unfolding also shape AGO2-target recognition^8^. Nevertheless, apart from the canonical, multiple non-canonical miRNA biogenesis pathways, using various combinations of the microprocessor, exportin, or miRISC loading complex proteins, have been reported^9,10^. In any case, AGO family is a prerequisite for the proper function of miRNAs.

In mammalian cells, AGOs are located and operate through canonical and non-canonical pathways both into the cytoplasm and nucleus^11^, in discrete foci^12–14^. However, the RISC can also reside/function in unexpected areas inside the cell, such as the GW-bodies, P-bodies and stress granules^15–17^, the midbody of dividing cancer cells^18^, along apical junctions^19^ with a putative tumor-suppressing function^20^ and in *Drosophila* nanotubes^21^. The GW-bodies most likely function as repositories for translationally silenced RNAs^17^, while inside the P-bodies or in the stress granules, AGO2 participates in miRNA-induced mRNA silencing^22^. In the apical zonula adherens, an alternative local regulation of selected miRNA processing was uncovered that suppresses the expression of markers critically involved in transformed cell growth^19^. Casey et al. reported the AGO2 localization in the midbody of dividing MCF10A, T47D, BT-474, and SK-BR-3 cells^18^. Furthermore, there has been an association between RISC and endosomes at synapses, through the interaction of PICK1 and AGO2, and the relocalization of the latter to endosomal compartment. This results in an elevated translation of mRNA targets locally^23^, dictating the spatio-temporal homeostasis. In conclusion, AGO2 most likely generates a niche creating the appropriate microenvironment locally for cellular and subcellular functions, maintenance, and regulation in a complex, multifaceted, and versatile manner.

In this article, we examined the AGO2 involvement in another locasome, the tubular protrusions, including tunneling nanotubes and cytokinetic bridges. Together, our results provide support for an AGO2 complex at the midzone, essential in cytokinesis and cell-division integrity.

## Results

### AGO2 resides in protrusional structures along with other components of the RNAi-machinery

**Fig. 1:**
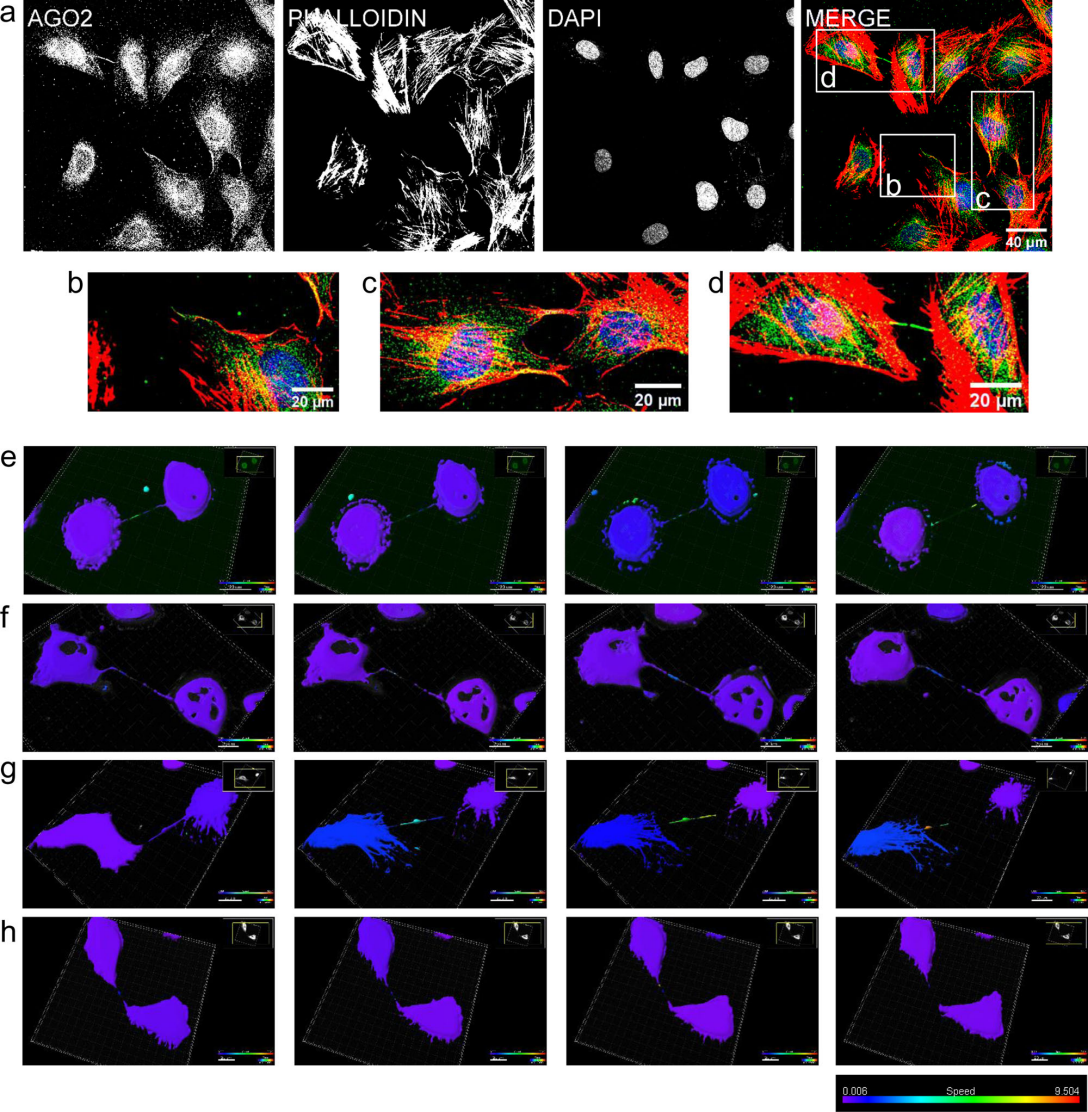
Subcellular distribution of AGO2. **a** Representative immunofluorescence images of the NTHY ori 3-1 cells (scale bar: 40 μm). Three types of AGO2 cellular protrusions are presented: **b** the typical Actin-filopodial structure (scale bar: 20 μm), **c** the open-ended tunneling nanotube (scale bar: 20 μm), and **d** the close-ended tubes (scale bar: 20 μm). AGO2 was depicted in green, F-Actin in red (Texas red-phalloidin), and nuclei in blue (DAPI). 3D surface reconstruction of time points from time-lapse experiments of AGO2-GFP expressing cells; **e**, **f** open-ended tubes and **g**, **h** close-ended tubes, processed by Imaris software. Surfaces are color-coded (blue to red) according to speed (low to high), showcasing the motility of the respective objects. The scale bar is 20 μm.

**Fig. 2:**
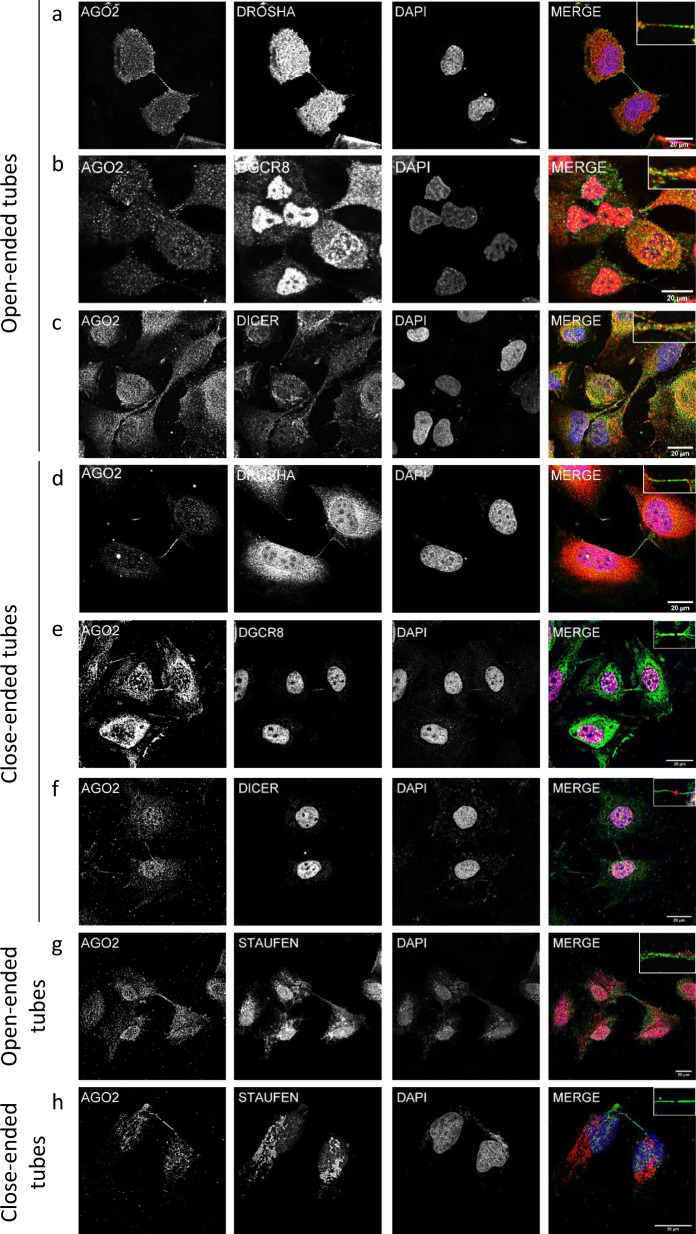
AGO2 resides in protrusional structures along with other components of the RNAi-machinery. Representative immunofluorescence images of NTHY ori 3-1 cells of **a**, **b**, **c**, **g** AGO2 open-ended tunneling nanotubes and **d**, **e**, **f**, **h** close-ended cellular protrusions with **a**, **d** Drosha, **b**, **e** Dgcr8, **c**, **f** Dicer, and **g**, **h** Staufen. AGO2 was visualized in green, Drosha, Dgcr8, and Dicer in red, and nuclei in blue (DAPI). The scale bar is 20 μm.

The AGO2 protein is located into the cytoplasm as well as into the nucleus of all the examined epithelial and mesenchymal cell lines, normal (thyroid NTHY ori 3-1, primary mammary HMEC-1 and hepatic stellate LX-2) and cancer (breast cancer MDA-MB-231, melanoma A375 and liver cancer HepG2), exhibiting a punctuate pattern (Fig. 1a and Supplementary Fig. S1a–f). Interestingly, AGO2 was also localized in protrusions, demonstrating differences in shape and density. More specifically, AGO2 resided in Actin-filopodial-like structures that are many per cell (Fig. 1a, b), and in tubular protrusions that are always formed in paired cells (Fig. 1a, c, d). The tubular protrusions demonstrate two different AGO2 distribution patterns, a low immunofluorescence AGO2 signal correlated with loosely shaped tunneling nanotubes, termed open-ended tubes (Fig. 1c), and an intense AGO2 signal in a compact and dense structure bearing a “gap” lacking AGO2, usually in the middle-line of the tube, termed close-ended tubes (Fig. 1d). To track the dynamic behavior of AGO2 in the two types of paired protrusions, time-lapse experiments were performed using cells expressing an AGO2-GFP chimeric protein. In the AGO2 tunneling nanotubes (open-ended), AGO2 components displayed flow and motility across the tube (Supplementary Movies 1 and 2 and Fig. 1e, f). AGO2 close-ended formations exhibited high flexibility, non-homogeneous structure, with a distinctively larger AGO2 object, when compared to open-ended nanotubes; the close-ended formation breaks, eventually leading to separation of the involved cells (Supplementary Movies 3 and 4 and Fig. 1g, h). Next, we investigated the presence and distribution of Drosha, DGCR8, and Dicer proteins, in the AGO2-enriched open- and close-ended paired structures. These crucial components of the RNAi-machinery functions, although they are known to reside mainly into the nucleus and cytoplasm, were also detected in the AGO2 tubes (Fig. 2a–f). Immunofluorescence assays demonstrated that Drosha and DGCR8 exhibited punctuate expression patterns within both tunneling open- (Fig. 2a, b) and close-ended nanotubes (Fig. 2d, e), similar to those observed with AGO2. Dicer was detected within and along the open-ended nanotubes (Fig. 2c), while in close-ended protrusions it was situated primarily in the middle-line (“gap”) of the tubes (Fig. 2f). Notably, Staufen, a double-stranded RNA (dsRNA) binding protein responsible for dsRNA transport and mRNA localization, was mainly cytoplasmic but was also found, though at lower signal intensities, inside the open-ended tunneling nanotubes (Fig. 2g), while being almost undetected in close-ended cellular protrusions (Fig. 2h). Altogether, it seems that in the open-ended tunneling nanotubes, ΑGΟ2 is loaded with dsRNA species, as indicated by the presence of Staufen. This does not seem to apply to the AGO2 close-ended protrusions, at least as suggested by the absence of Staufen. Given the strong signal of AGO2 in the close-ended structures and the distinct pattern with the “gap” in the middle of the tube, their composition and functional contribution to cellular physiology needs to be further elucidated.

### AGO2 close-ended protrusions constitute a different type than typical Actin-filopodial structures

To obtain more information regarding the structure of AGO2 close-ended protrusions we compared them to the well-established Actin-filopodial-like ones. A total of 168 confocal laser scanning microscopy (CLSM) images of individual cells (Supplementary Figs. S2a, b and S3a, b), from at least three independent biological experiments, were collected and processed. The number of protrusions per cell, their lengths, and the tip-to-midpoint width of both types of structures were measured. The statistical analysis of the measurements revealed significant differences that clearly distinguish between the two types of protrusions. In all, 38 Actin-filopodial protrusions on average were present per cell, whereas only one AGO2 protrusion could be maximally identified per cell. Intriguingly, although the length of Actin-filopodial protrusions ranges from 0.48 to 37.76 µm with a mean of 2.85 μm, the AGO2 close-ended protrusions exhibit less divergence (0.88–19.00 µm) and their mean length was significantly (*p* = 2.2e^−16^) larger (5.37 μm) (Supplementary Fig. S2c). In an attempt to obtain a model shape of the structures, the average tip-to-midpoint width ratio of all protrusions was calculated. The average ratio of AGO2-carrying structures (1.03 μm) differed significantly (*p* = 7.58e^*–*07^, Supplementary Fig. S2d) from the typical triangular shape of Actin-filopodial protrusions (mean ratio 0.85 μm), showing a tendency toward a parallelogram-like formation (Supplementary Fig. S2e). In conclusion, AGO2 close-ended protrusions differ from the typical Actin-filopodial ones as they are infrequent, parallelogram-shaped, and always formed in paired cells.

### AGO2 close-ended cellular structures are cytokinetic protrusions

**Fig. 3:**
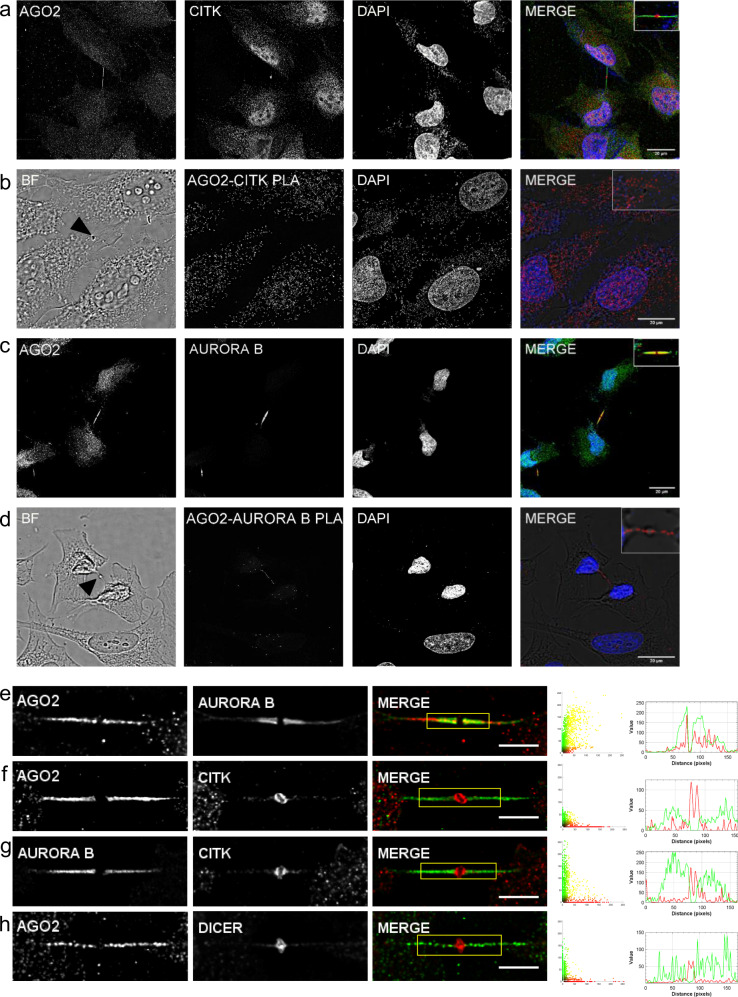
AGO2 close-ended cellular structures are cytokinetic protrusions. Representative immunofluorescence images of NTHY ori 3-1 cells depicting the subcellular localization of AGO2 (green) with **a** Citron kinase (CITK) (red) and **c** Aurora B (red). Cell nuclei were stained blue (DAPI). **b**, **d** iPLA assays depicted the proximity of **b** AGO2–CITK and **d** AGO2–Aurora B as indicated by red foci. The black arrowheads in the Brightfield (BF) images indicate the midbody ring. The scale bar is 20 μm. Visual assessment (merged images, scatterplots, and intensity lineplots) of colocalization between paired proteins included in the yellow region of interest of the merged images. **f**, **h** AGO2 (green) with CITK and Dicer (red), respectively. **e**, **g** Aurora B in green with AGO2 and CITK in red, respectively. Lineplots indicate the signal intensity of the paired proteins along the lines overlaid on the corresponding images that appear in Supplementary Fig. S4d–g. The colocalization metrics (Pearson [*R*] and Manders’ [*M*1 and *M*2] coefficients) and respective *p* values are presented in Supplementary Table S1 (*n* = 6 biologically independent samples). Scale bar: 5 μm.

Based on the aforementioned findings, we investigated the possibility of the close-ended protrusions to comprise midbody structure, a narrow intercellular bridge arose during cytokinesis, the final step of cell division. To explore this scenario, we examined the presence of cytokinesis-related proteins in these structures. Citron kinase (CITK), a protein carrying a coiled-coil domain that differentially dictates its subcellular localization and leads to proper midbody stabilization during cytokinesis, was detected in the close-ended AGO2 protrusions. CITK was primarily enveloped at the area called midbody ring (Fig. 3a) that in the close-ended protrusions AGO2 signal is absent and thus depicted as “gap.” In situ proximity ligation assay (iPLA) experiments of AGO2 and CITK demonstrated the accumulation of fluorescent spots to be restricted into the cytoplasm and almost absent along the intercellular bridge (Fig. 3b). In contrast, Aurora B, the other critical cell-division kinase that is essential for the functional midbody architecture through CITK cross-regulation, exhibited a striking overlap with AGO2 (Fig. 3c). The AGO2–Aurora strong correlation was also confirmed with the iPLA experiments (Fig. 3d). Colocalization analysis (Fig. 3e–h and Supplementary Fig. S4d–g) and corresponding statistical tests strongly supported these findings (Supplementary Table S1). In particular, the CITK–AGO2 pair exhibits low *M*2 value—proportion of CITK channel occupying pixels of the AGO2 channel—(*M*2 = 0.336), since CITK is primarily detected at the midbody ring whereas AGO2 is absent (Fig. 3e). On the contrary, strong colocalization emerged between AGO2 and Aurora B (*R* = 0.718, *M*1 = 0.889, *M*2 = 0.786), as Aurora B resided almost entirely along the intercellular bridge being occupied by AGO2 (Fig. 3f). CITK and Aurora B demonstrated differential distribution, in complementary patterns, in the AGO2 close-ended cellular structures (Fig. 3g). Finally, Dicer was primarily located at the midbody ring (Supplementary Fig. S4a–c, h, l, m), as indicated by the intensity plots (Fig. 3h). The presence of CITK and Aurora B kinases in the AGO2 close-ended cellular structures revealed that these comprise cytokinetic protrusions.

### *α*-Tubulin alterations and cytoskeletal changes influence AGO2 localization

**Fig. 4:**
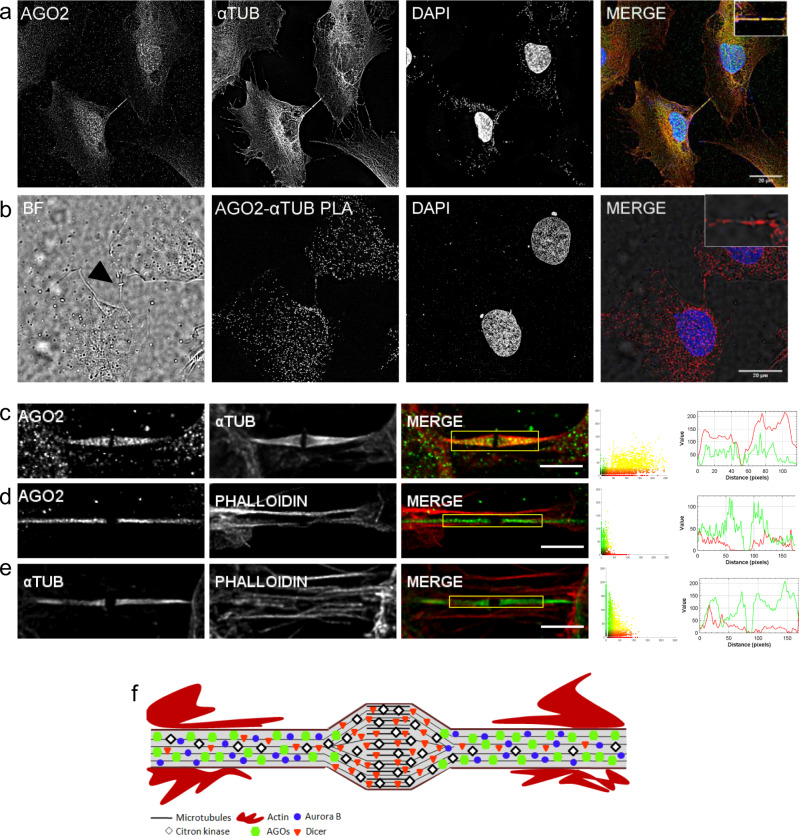
AGO2 colocalizes with *α*-Tubulin in mitotic protrusions. **a** Representative immunofluorescence images of NTHY ori 3-1 cells depicting the colocalization of AGO2 (green) with *α*-Tubulin (red). Cell nuclei were stained blue (DAPI). **b** iPLA assays demonstrated the high proximity of AGO2 and *α*-Tubulin by the presence of red foci. The black arrowhead in the BF images indicates the midbody ring. The scale bar is 20 μm. **c**–**e** Visual assessment (merged images, scatterplots, and intensity lineplots) of colocalization. Lineplots indicate the signal intensity of the paired proteins along the lines overlaid on the corresponding images that appear in Supplementary Fig. S4i–k. The colocalization metrics (Pearson [*R*] and Manders’ [*M*1 and *M*2] coefficients) and respective *p* values are presented in Supplementary Table S1. **c**, **d** AGO2 in green, *α*-Tubulin, and F-Actin (Texas red-phalloidin) in red. **e** *α*-Tubulin in green, F-Actin in red (*n* = 6 biologically independent samples). **f** Diagrammatic representation of the components of the cytokinetic intercellular bridge including AGO2, Dicer, CITK, Aurora B, *α*-Tubulin, and F-Actin. Scale bar: 5 μm.

Given that AGO2 appears to reside in cytokinetic protrusions, the possibility of a tight configuration with *α*-Tubulin polymers (microtubules) was investigated. Polymerized *α*-Tubulin was strongly colocalized with AGO2 in the emerged blebs of cytokinesis and it was absent from the ring of midbody formation (Fig. 4a, c and Supplementary Fig. S4i). This was also demonstrated through iPLA assays with multiple foci being observed along the midbody arms, but not at the midbody ring (Fig. 4b). However, F-Actin (G-Actin polymer), the other fundamental cytoskeleton component, was presented with significantly lower degree of colocalization with AGO2 (Supplementary Table S1). Actin filaments were mainly located close to the starting point of the protrusions and were attenuated along the intercellular bridge (Fig. 4d, e and Fig. S4j, k). A representation of the distribution patterns of all the herein examined proteins (AGO2, Dicer, CITK, Aurora B, *α*-Tubulin, and F-Actin) across the intercellular bridge is illustrated in Fig. 4f.

**Fig. 5:**
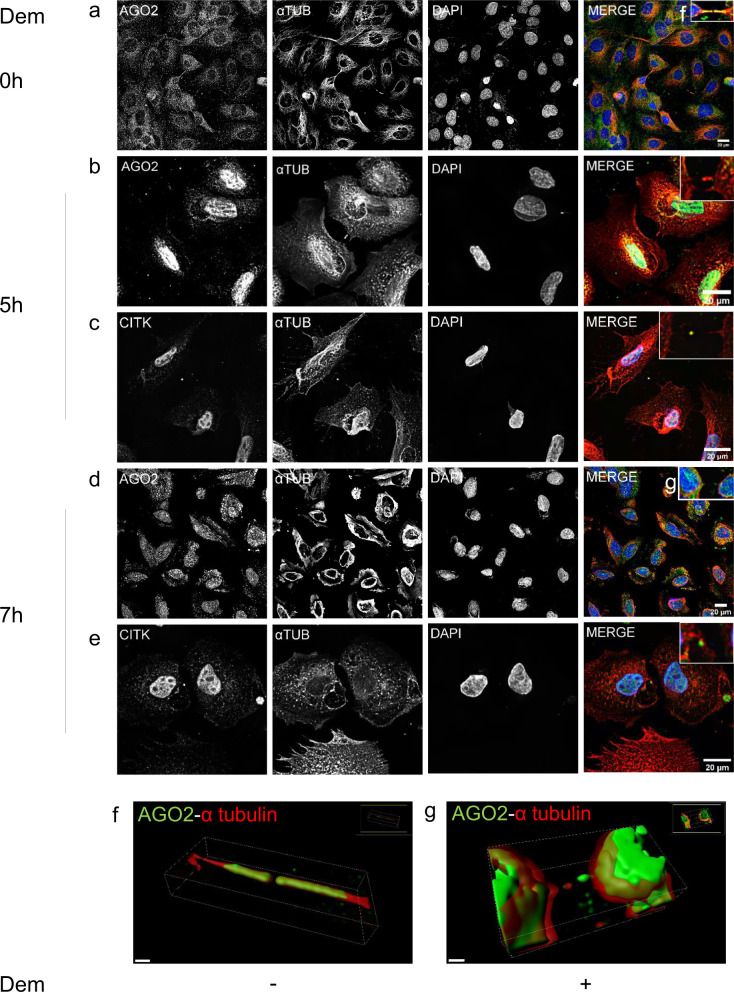
*α*-Tubulin alterations and cytoskeletal changes influence AGO2 localization. Representative images of NTHY ori 3-1 cells **a** before treatment with Demecolcine (Dem) and at **b**, **c** 5 h, and **d**, **e** 7 h after treatment. **a**, **b**, **d**, **f**, **g** AGO2 was stained green, **a**–**g** *α*-Tubulin red and **c**, **e** CITK green. Nuclei of cells were visualized in blue (DAPI). The scale bar is 20 μm. 3D surface reconstruction by Imaris of the cytokinetic bridges **f** before (scale bar: 15 μm) and **g** after Demecolcine treatment (scale bar: 10 μm).

**Fig. 6:**
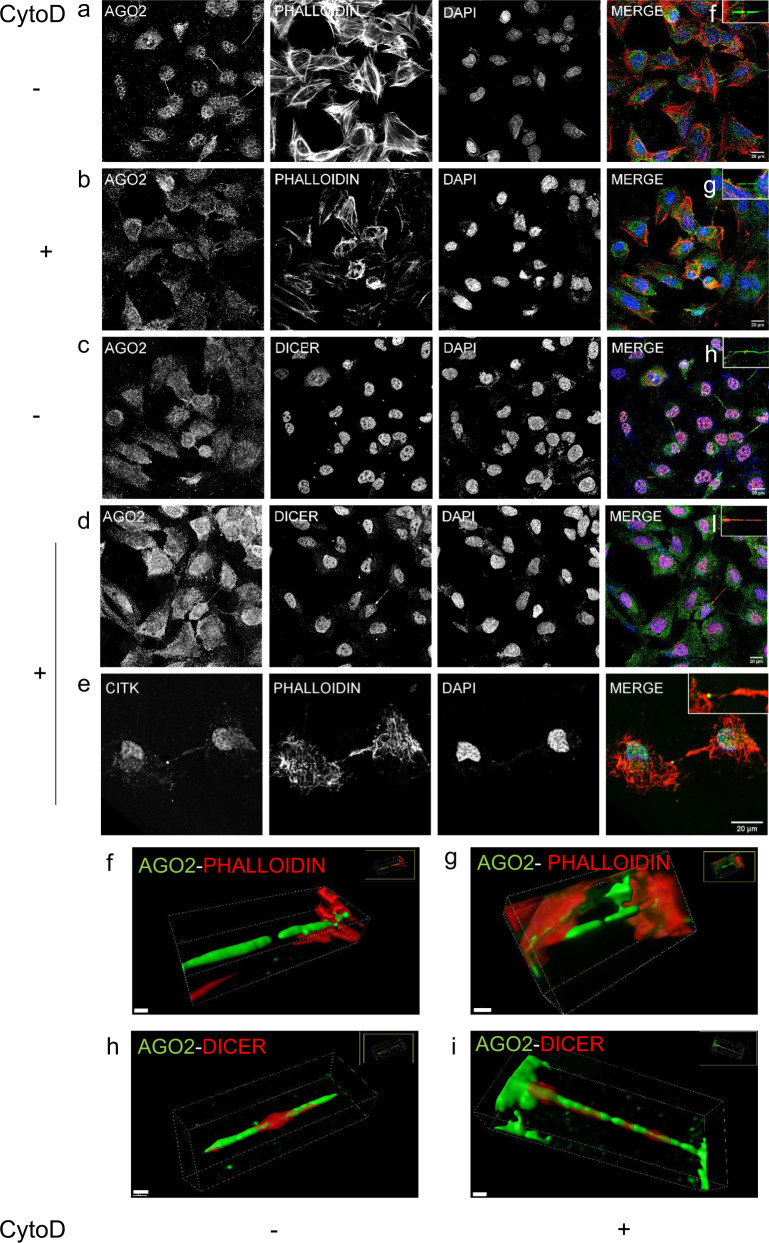
Dicer distribution associated with F-Actin. Representative images of NTHY ori 3-1 cells **a**, **c** before and **b**, **d** after Cytochalasin D (Cyto D) treatment. **a**–**d**, **f**–**i** AGO2 in green, **a**, **b**, **e**–**g** F-Actin in red, **c**, **d**, **h**, **i** Dicer in red and **e** CITK in green (scale bar: 20 μm). Nuclei of cells were visualized by blue staining (DAPI). 3D surface reconstruction by Imaris of the cytokinetic bridges **f**, **h** before (scale bar: 15 μm) and **g**, **i** after Cyto D treatment (scale bar: 3 and 10 μm, respectively).

We further investigated the relationship of AGO2 with the two pivotal components of cytoskeleton, *α*-Tubulin and F-Actin, in a drug-induced depolymerization manner. To this end, Demecolcine and Cytochalasin D, specific inhibitors targeting the microtubule and Actin-filament depolymerization, respectively, were applied and their effects on cell morphology, AGO2 distribution, and formation of cytokinetic and cytoplasmic protrusions were examined. After 5 h of Demecolcine treatment, *α*-Tubulin was not totally depolymerized across the intercellular bridges and AGO2 exhibited decreased signal intensity (Fig. 5a, b). The midbody formation was not completely disrupted as indicated via the normal distribution of CITK (Fig. 5c). Most importantly, after 7 h of Demecolcine treatment, a depolymerization of intercellular bridges was induced as indicated by the disruption of *α*-Tubulin structures, with a similar fragmentation of AGO2 protrusions (Fig. 5d, f, g). The AGO2 expression signal was directed toward the cytoplasm at lamellipodia and in membrane ruffles (Fig. 5d). However, the localization profile of CITK clearly points toward an intact midbody ring formation (Fig. 5e). In conclusion, AGO2 distribution is related to the intact polymerization of *α*-Tubulin, indicating that *α*-Tubulin can constitute a capable scaffold for AGO2 transfer and function (Fig. 5f, g). Exposure to Cytochalasin D did not cause detectable changes in AGO2 typical distribution (Fig. 6a, b, f, g). Nevertheless, Cytochalasin D prevented Dicer accumulation in the midbody and compelled, in some cases, its distribution along the AGO2 midbody arms (Fig. 6c, d, h, i). Remarkably, the localization pattern of CITK remained unaffected, hence, indicating the specificity and component-selectivity of the drug-induced molecular phenotype. (Fig. 6e). Dicer-specific foci, frequently observed inside the cytoplasm and in the milieu, proved to serve as markers of midbody remnants after abscission completion (Fig. 6c). It seems that the scattering of AGO2 and Dicer is likely dictated by different regulatory mechanisms.

### AGO2 follows the *α*-Tubulin cellular expression patterning during cell division

As the strong correlation between AGO2 and *α*-Tubulin was already demonstrated, it is not surprising that AGO2 follows the *α*-Tubulin cytoskeleton distribution pattern during the different phases of cell cycle (Supplementary Fig. S5). In particular, in metaphase that is typified by chromosomes being aligned during central spindle assembly, AGO2 showed a dense cytoplasmic signal with the exception of the area in-between the chromosomes. However, AGO2 and Dicer immunofluorescent topologies, following the mitotic spindle, become concentrated at the mitotic poles (Supplementary Figs. S5a and S6a). In anaphase, AGO2 was highly accumulated in the centrosomal area (Supplementary Fig. S5b), while Dicer followed the microtubule-segregation (Supplementary Fig. S6b). In this phase, the initial cue for cytoskeleton rearrangement, the creation of microtubule-bundling area called midzone, and the cleavage furrow ingression become readily recognizable. In telophase, AGO2-localization pattern demonstrated a “gap” in the middle of the midzone area, similar to the *α*-Tubulin configuration (Supplementary Fig. S5c). In parallel, Dicer was mainly located at the midbody ring (Supplementary Fig. S6c). The intercellular bridge starts its formation during late telophase, with a simultaneous compression of midzone and the development of microtubule midbody. AGO2 and Dicer reside in the intercellular bridge; during the process of stabilization of cleavage furrow ingression AGO2 formed a tight arrangement inside and along the *α*-Tubulin bridge (Supplementary Fig. S5d), while Dicer was mainly distributed around the ring and gradiently decreased inside the arms (Supplementary Fig. S6d). Then, the cleavage furrow begins the narrowing until the intercellular bridge is disintegrated and the two daughter cells are emancipated (Supplementary Figs. S5e and S6e). Following the *α*-Tubulin constriction and abscission, AGO2 regressed toward the daughter cells following the midbody together with the arm-structure into the cell cytoplasm for autophagy-mediated elimination (Supplementary Fig. S5f). The Dicer-labeled midbody ring was digested from one cell in the case of asymmetric division or removed to the cell milieu in case of symmetric division (Supplementary Fig. S6f). Altogether, the AGO2 expression pattern during mitosis and its resemblance to the -polymerized- *α*-Tubulin respective one reinforce the notion that AGO2 sustains important roles in cell division.

### AGO2 over-expression generates aneuploidy via cytokinesis errors

**Fig. 7:**
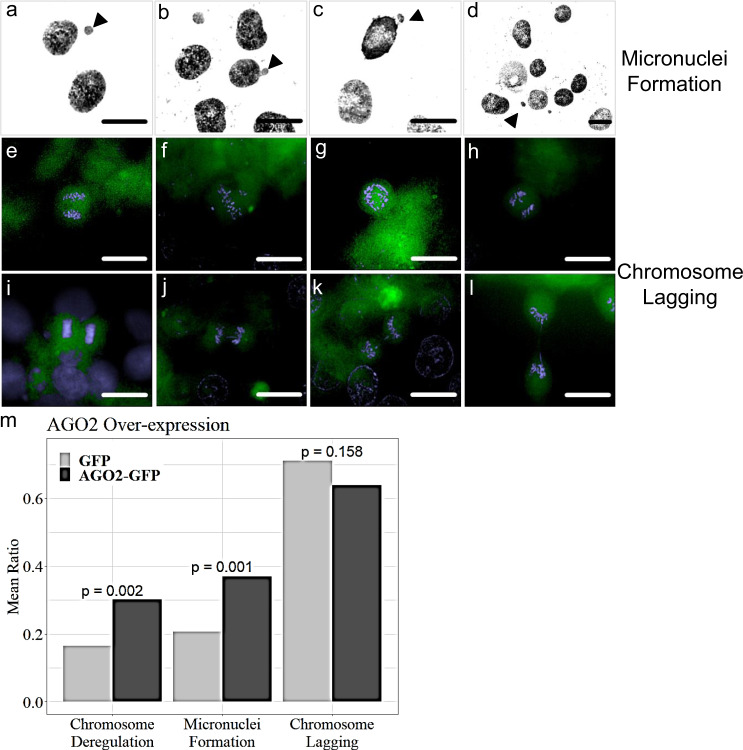
AGO2 over-expression leads to numerical chromosomal instability and micronuclei formation. **a**–**d** Representative images of HCT116 AGO2-GFP transfected cells depicting **a**, **c**, **d** micronuclei formations and **b** bleb nuclei, as indicated by the black arrowheads. **e**, **i** normal anaphase and cytokinesis. AGO2-GFP transfected cells carry **f** abnormal metaphase congression, **g** anaphase or **j** cytokinetic chromosomal bridges, and **h** lagging chromosomes in telophase or **k**, **l** in cytokinesis. GFP expression in green and nuclei in blue (DAPI) (scale bar: 10 μm). **m** Bar plot of the effects of AGO2 over-expression on numerical chromosome deregulation, micronuclei formation, and lagging chromosomes. *z*-test was used to evaluate their significance. Bar height indicates the ratio of deregulated chromosomes (*n* = 3 biologically independent experiments, statistical sample size *n*1 = 188 GFP cells, *n*2 = 182 AGO2-GFP cells), cells with micronuclei formation (*n* = 3 biologically independent experiments, statistical sample size *n*1 = 174 GFP cells, *n*2 = 168 AGO2-GFP cells), and with lagging chromosomes (*n* = 3 biologically independent experiments, statistical sample size *n*1 = 176 GFP cells, *n*2 = 169 AGO2-GFP cells), respectively.

To further study the contribution of AGO2 to the fidelity of cell division, we examined the effect of over-expression and downregulation of AGO2 in chromosome segregation. HCT116 cells were transfected to transiently express AGO2-GFP. The AGO2 over-expression induced statistically significant micronuclei formation (Fig. 7a–d, m) and numerical chromosomal deregulation (gain or loss) (Supplementary Fig. S7a–c and Fig. 7m). However, although there were detected lagging chromosomes in anaphase and cytokinesis (Fig. 7e–l), the percentage was similar to the control, an observation that needs further investigation. These findings lead to the conclusion of AGO2 implication in aneuploidy, reflecting post-replicative events and cytokinesis errors. By knocking down AGO2 (almost two-fold-changes) (Supplementary Figs. S7h and S8a), no statistically significant changes in numerical chromosomal deregulation and micronuclei formation were observed, but structural alterations emerged (Supplementary Fig. S7d–g). Cytokinesis-failure events such as binuclear cell formations and midbody abnormalities such as double midbody rings (Supplementary Fig. S7i–k) demonstrate that AGO2 influences the integrity and the succession of cytokinesis. Altogether, these results illustrate the implication of AGO2 in fine-tuning chromosomal regulatory mechanisms, safeguarding the cell-division success.

### Kinases follow the AGO2/Dicer distribution in the midbody structure

**Fig. 8:**
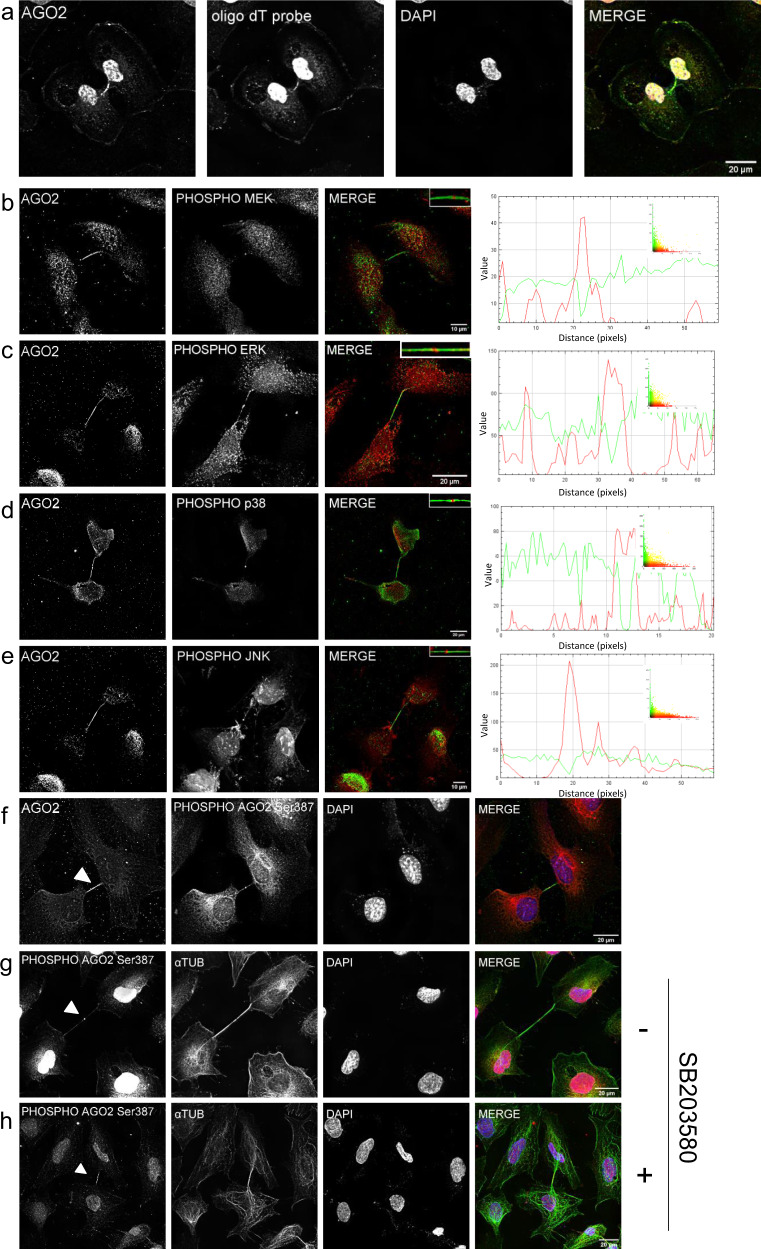
Activated kinases follow the AGO2/Dicer distribution in the midbody structure. Representative images of NTHY ori 3-1 cells. AGO2 colocalization with **a** biotinylated oligo dT probe (scale bar: 20 μm). Small kinome analysis of **b** AGO2-phospho-MEK (scale bar: 10 μm), **c** AGO2-phospho-ERK (scale bar: 20 μm), **d** AGO2-phospho-p38 (scale bar: 20 μm), **e** AGO2-phospho-JNK (scale bar: 10 μm). **a**–**e** AGO2 proteins were visualized in green, οligo dT probe, phospho-MEK, phospho-ERK, phospho-p38, and phospho-JNK were visualized in red. **b**–**e** Visual assessment (merged images, scatterplots, and intensity lineplots) of colocalization. **b**–**e** Lineplots indicate the signal intensity of the two paired proteins. Images of AGO2 colocalization with **f** phospho-AGO2 Ser^387^ and phospho-AGO2 Ser^387^ with *α*-Tubulin in **g** untreated and **h** treated cells with SB205380 p38 inhibitor. **f**–**h** Phospho-AGO2 Ser^387^ in red, **f** AGO2 and **g**, **h** *α*-Tubulin in green, respectively. Nuclei were visualized in blue (DAPI). Scale bar: 20 μm.

The role of AGO2 in the midbody might be an unrecognized contribution to cell division or related to a function of the known -typical- AGO2 pathway. In an attempt to distinguish between the two scenarios and better understand AGO2 involvement, we herein investigated the presence of poly-A^+^ tailed transcripts along the cytokinesis bridge (Fig. 8a). Surprisingly, the hybridization (oligo dT) probe followed the AGO2 pattern. This finding together with the complementary Dicer distribution pattern (Fig. 3h) indicated a Dicer-independent AGO2 function in the arms of the midbody structures.

However, as the lack of AGO2 detection from the midbody ring was striking, we further investigated the possibility that post-translationally modified AGO2 protein molecules also resided at this site. Phosphorylation is a crucial regulation step of AGO2 activation^24^ and, therefore, we explored the occurrence of different activated kinases in the cytokinetic bridge during the late steps of cell division. Colocalization analysis of a small kinome including phospho-MEK (Fig. 8b), phospho-ERK (Fig. 8c), phospho-p38 MAPK (Fig. 8d), phospho-JNK (Fig. 8e), phospho-Akt (Supplementary Fig. S9a), and phospho-AMPK (Supplementary Fig. S9b) showed that there are two distinct, almost complementary to each other, patterns in the cytokinetic structure. Phospho-MEK, -ERK, -p38, and -JNK, although being present along the midbody arms, were highly concentrated at the ring following Dicer’s distribution, while phospho-Akt (Supplementary Fig. S9a) and phospho-AMPK (Supplementary Fig. S9b) occupied the cytokinetic protrusions leaving a small “gap” at the midbody ring resembling the AGO2 pattern. The specificity of these distribution patterns is enhanced by the fact that other cell cycle-specific kinases such as phospho-CHK2 were absent across the cytokinetic bridge (Supplementary Fig. S8b). Due to the midbody distribution of phospho-MEK/ERK/p38/JNK and the fact that the p38 MAPK pathway enhances the phosphorylation of AGO2 at the catalytic residue Serine ^387^, we investigated the presence of phospho-AGO2 Ser^387^ in the midbody. Interestingly, the phospho-AGO2 Ser^387^ follows the phospho-MEK/ERK/p38/JNK pattern, exhibiting high concentration in the midbody ring (Fig. 8f). To strengthen the p38-dependent AGO2 phosphorylation in the bridge, we treated the cells with SB203580, a selective inhibitor of p38 MAPK. Indeed, the phospho-AGO2 Ser^387^ was relocated in a number of cytokinetic bridges, moving from the ring to the arms of the midbody (Fig. 8g, h). Ιn conclusion, the phospho-AGO2 Ser^387^ is located in the ring of the midbody in a p38 MAPK-dependent manner. The presence of Dicer in the same locus suggests an active RISC machinery in a non-expected location, thus implying an alternative function of the complex during cell division.

### ΑGO2 is a stress-sensitive molecule

**Fig. 9:**
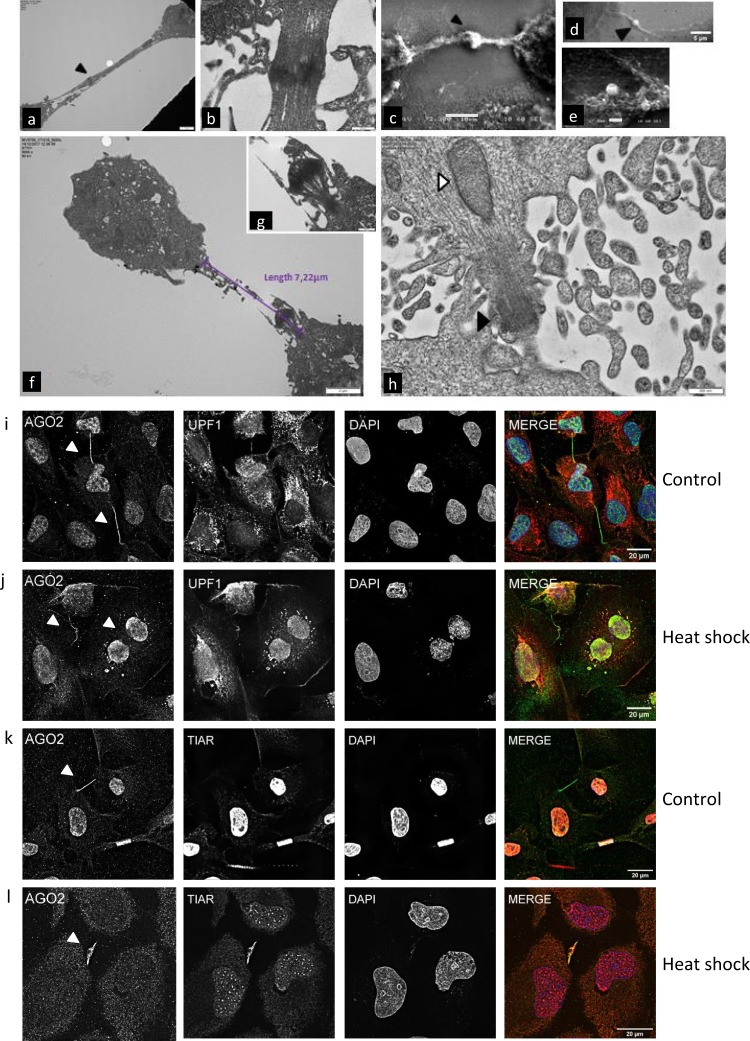
ΑGO2 is a stress-sensitive molecule. Electron microscopy images of the stages of cell division of NTHY ori 3-1 cells depicting the cytokinetic structures up to abscission. **a**, **b**, **f**–**h** Section transmission electron micrographs and **c**–**e** scanning electron micrographs of dividing cells. **a** Dense bundled microtubules (black arrowhead) in the midzone and **b** a magnified midzone. **c**, **d** The midbody core and the electron-dense material (flemming body) concentrated at the midzone. **e** Cargoes are transferred through the microtubule protrusions. **f**, **g** A discontinued intercellular bridge between two daughter cells after abscission. The nucleus of the left-hand cell is visible. **g** A magnified abscission point. **h** The characteristic structural architecture of microtubular axoneme after the completion of abscission (black arrowhead). Membrane deposition is pictured by the black arrowhead. Scale bars: **a** 2 μm, **b** 500 nm, **c** 10 μm, **d** 5 μm, **e** 2 μm, **f** 2 μm, **g** 500 nm, and **h** 500 nm. **i**–**l** Representative images of NTHY ori 3-1 cells before and after heat shock. AGO2, Upf1, and TIAR subcellular distribution in **i**, **k** control and **j**, **l** heat-shocked cells. AGO2 is represented in green, Upf1, TIAR in red, and nuclei in DAPI (blue). The white arrowheads indicate cytokinetic events (scale bar: 20 μm).

The above findings support the strong implication of AGO2 in successful cell division. The dividing cells are known to be under tremendous stress^25^, a finding corroborated by the existence of the abovementioned phospho-kinases locally. As the role of kinases in mechanical stress has been previously reported^26^, the presence of several activated kinases in the midbody/midzone area is likely associated with the development of locally applied stress, during late cell division. To support this, we conducted transmission electron microscopy (TEM) and scanning electron microscopy (SEM) high-resolution technologies. A proteinaceous electron-dense material was observed at the midzone of microtubule bundles with the typical distance being highly varied (Fig. 9a–g). In SEM images, light-colored, electron-dense material was apparent (Fig. 9c, d). This material, likely containing molecular cargoes and proteins, circuited at the protrusions providing views of rough surfaces (Fig. 9e). When abscission occurs (Fig. 9f, g) the midbody is withdrawn and uptaken/fused with one daughter cell for digestion. Notably, dividing cells did not carry filopodial protrusions anti-diametrically of the intercellular bridge (Fig. 9f), indicating that during the exertion of mechanical forces -polymerized- *α*-Tubulin and F-Actin cytoskeleton, together with membrane depositions, are rearranged for the formation of midzone arms and midbody ring. After abscission mitotic arm protrusions become contracted (Fig. 9f) and vesicle inventories (Fig. 9h) are conspicuous, probably for membrane deposition and remodeling. Since the dividing cells are under tremendous stress and AGO2 appeared to carry a functional role in cell division, we reasoned that AGO2 is a stress-sensitive molecule. Indeed, heat-shock treatment malformed or abolished AGO2 close-ended protrusions (Fig. 9i–l). The finding was strengthened by noteworthy changes in colocalization of AGO2 with the stress-related proteins, Upf1 and TIAR. In particular, under local basal stress, Upf1 and TIAR proteins were detected at the midzone area and their granules were weakly colocalized with AGO2 protein (Fig. 9i, k). After heat shock, the AGO2 foci were colocalized with Upf1 stress granules and the malformed cytokinetic bridges, wherever they developed, demonstrated a strong colocalization pattern with the Upf1 and TIAR respective ones (Fig. 9j, l). These findings supported the stress-sensitive nature of AGO2 that was accompanied by cytokinesis-failure events.

**Fig. 10:**
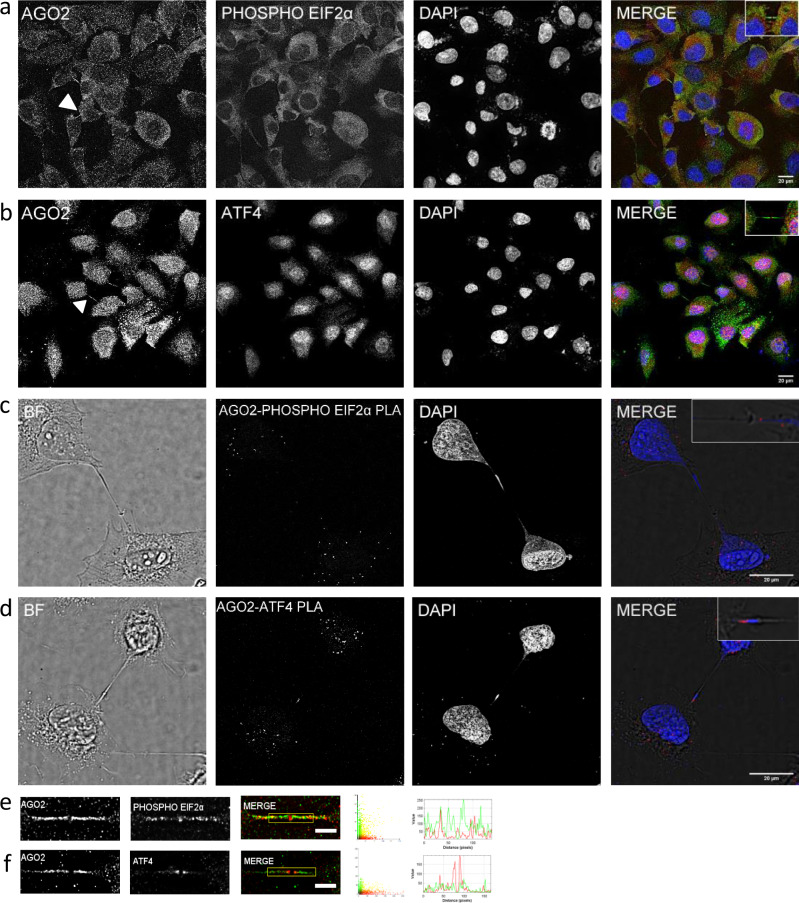
Subcellular localization and interaction between AGO2 and ER-stress regulators. Representative images of NTHY ori 3-1 cells showing AGO2 colocalization with **a** phospho-Elf2α and **b** ATF4. AGO2 proteins in green, phospho-EIf2α and ATF4 in red. Nuclei of cells were visualized in blue (DAPI). iPLA foci between **c** AGO2-phospho-EIf2α and **d** AGO2-ATF4 in red. **e**–**f** Visual assessment (merged images, scatterplots, and intensity lineplots) of colocalization. Lineplots indicate the signal intensity of the paired proteins along the lines overlaid on the corresponding images that appear in Supplementary Fig. S2e–f. Scale bar: 20 μm (full images), 5 μm (zoomed images).

We further investigated the implication of AGO2 to a specific type of stress, ER-stress, which is required for a successful cytokinesis^27^. ER-stress modulated effectors such as eIF2α translation factor and its downstream target, *ATF4*, are found along the intercellular bridge and into the midbody ring (Fig. 10a, e and Supplementary Fig. S4n, o). iPLA foci of AGO2-phospho-eIF2*α* and AGO2-ATF4, although located into the cell matrix, were also sparsely located within the bridge (Fig. 10c, d) indicating that AGO2 may trigger ER-stress effectors for ATF4 activation. The interaction foci were detected in both sides of the bridge likely implying a cytoprotective regulation of local-type transcript homeostasis of gene expression. The coexistence of the four activated phospho-kinases, MEK, ERK, p38, and JNK, together with the phospho-eIF2*α* and ATF4 proteins into the AGO2-enriched cytokinetic structures strongly suggests the simultaneous and synergistic activation of the MAPK-signaling network and RNAi-machinery in response to stress (mechanical and/or ER), in a topology-specific manner, most likely for the restoration of local -transcript- homeostasis during cytokinesis.

## Discussion

AGO2, a protein of the miRISC machinery, catalyzes the mRNA degradation or translational repression in the cytoplasm, through guidance by miRNAs that are loaded on AGO2 complexes. Nevertheless, AGO2 can also reside into the nucleus, acting in a typical RNAi manner^28^. Alternative splicing process, RNA-mediated epigenetic regulation through RISC-chromatin interactions and double strand-break repair are among the emerging nuclear functions of AGO family members^28–33^. In mammalian cells AGOs are located and operate through canonical and non-canonical pathways both into the cytoplasm and nucleus^11^ in discrete foci^12–14^ in physiological and pathological conditions^34^. RISC can also function in GW-bodies, P-bodies, and stress granules^15–17^, along apical junctions^19^ and in *Drosophila* nanotubes transmitting intercellular signals^21^, regulating translation of mRNA targets locally^23^ and therefore dictating the spatio-temporal homeostasis. In addition to the above, we herein provided evidence of an AGO2-niche in membrane protrusions, suggesting its critical role(s) in these structures. Specifically, we demonstrated the accumulation of AGO2 in open-ended tunneling nanotubes and close-ended cytokinetic protrusions, in human cells.

Human cellular protrusions are known to be involved in intercellular trafficking of cytoplasmic or genetic material^35,36^, pathogen transmission^37,38^, mitochondria, calcium, and other cargoes transportation^35,39^, and miRNA and protein communication^40^. In dendritic spines, AGO2 constitutes an important element for derepression of dendritic mRNAs and local protein synthesis triggered by synaptic activity, thus introducing the concept of spatio-temporal participation of AGO2 in local protein regulation^41^. Our findings support a similar AGO2-dictated local regulation in other cell types such as epithelial cells. In our experimental setting the AGO2-niche correlated with loosely shaped tunneling nanotubes termed open-ended tubes. Drosha, DGCR8, and Dicer, crucial components of the miRNA machinery, as well as Staufen, a dsRNA-binding protein, were also shown to reside into these nanotubes. Live imaging demonstrated the AGO2 protein motion inside the nanotube. Combined with the presence of the abovementioned proteins, these loci indicate RNAi-machinery motility in these structures. Through this action, cells can conduct intermolecular cross-talking, exchange RNA signals, and regulate transcript instability (half-life time) or translational repression at a very specific local (micro-) environment.

In the case of AGO2 close-ended protrusions, alternative regulatory functions are likely engaged. The Dicer pattern, the detected Staufen protein, and the presence of oligo dT probe at the intercellular bridge indicated alternative, non-typical AGO2 functions, probably deployed in a miRISC-independent manner. The AGO2 close-ended tubes were different structures from the Actin-filopodial ones, as they were unique per cell, parallelogram-shaped, and always appearing in paired cells. The detection of CITK and Aurora B kinases in these cellular structures confirmed that they comprise bona fide cytokinetic protrusions (Aurora B regulates cytokinesis through microtubule interactions and stabilization of microtubule structures^42,43^). Stably growing and rapidly shrinking microtubules actively participate in cytoskeletal remodeling, delivering dramatic cell changes^44^. AGO2 is present in a tight configuration along *α*-Tubulin polymers (microtubules) into the intercellular bridges of cytokinesis. Although it resided in extremely high concentrations in the midbody arms, also reported by Casey et al. in 2019^18^, it leaves an empty space, a “gap,” in the area of midbody ring. *α*-Tubulin distribution in cells exposed to Demecolcine, a specific inhibitor of tubulin polymerization, was disorganized and AGO2 protrusions were forced to fragmentation and AGO2 is concentrated at lamellipodia. This indicates that *α*-Tubulin may serve as a major scaffold for AGO2 local motility. On the other hand, Dicer, highly accumulated in the midbody ring, was significantly affected by F-Actin. Architectural derangement of F-Actin induced by Cytochalasin D cell exposure prevented Dicer accumulation in the midbody and occasionally compelled it in unexpected areas along AGO2 arms. The above indicate the differential regulation of AGO2 and Dicer and reinforced the notion that AGO2 sustains an important role in cell division.

AGO involvement in chromosome division phenomena has been previously reported in a number of model organisms such as yeast, *Drosophila*, and mouse. TbAGO1 is known to be crucial for mitotic spindle assembly and chromosome segregation in the *Trypanosome brucei* parasite^45^. In yeast deletion of *Dicer1* or *AGO1* was found to disrupt chromosome segregation, leading to chromosome lagging and centromere-silencing abrogation^46^. In *Drosophila melanogaster* epigenetic gene silencing modulation and cytokinesis are regulated via the interaction of AGO1-sticky/CITK^47^. In case of meiosis the germ cell-specific rice gene *MEL1*, an AGO family member in plants that controls the division of pre-meiotic germ cells, properly modifies the meiotic chromosomes^48^. In mice AGO4 is implicated in the precise chromosome segregation and is required for the proper entry into meiosis in germ cells^49,50^. In addition, Dicer can prevent mitotic defects during meiosis in mouse oocytes^51^. In mammals AGO2 catalytic activity, but not the classical miRNA pathway, is crucial for proper chromosome segregation. Moreover, Dicer generates ASAT (α-satellite) siRNAs, which, synergistically with AGO2, control satellite RNAs^52^. These reports support the mechanistic association of AGO2 with cell-division machinery in a wide evolutionary spectrum of organisms, including humans. Our findings underpin this association, since over-expression of *AGO2* results in a numerical deregulation (gain, or loss) of chromosomes. Moreover, these cells present statistically significant micronuclei formation, providing evidence for aneuploidy events. Downregulation of *AGO2* induces a number of abnormalities, such as binuclear formations, blebs, double midbody rings, and structural chromosomal instabilities, thus indicating the indispensable contribution of AGO2 to the regulation, progression, and success of cytokinesis.

As mitosis requires high cytoskeletal rearrangements and continuous supply of energy and biomolecules for its successful and unimpaired implementation, the presence of kinases such as phospho-MEK, -ERK, -JNK, -p38, -Akt, and -AMPK in the cytokinetic bridge was not unexpected. Previous studies have highlighted the crucial regulatory roles of the herein examined kinases in the midbody formation and cytokinesis^53–56^, with their contribution to cell division mostly being manifested through activation of critical downstream targets for regulating microtubule dynamics and F-Actin cytoskeletal integrity^57^. In our experiments phospho-Akt and -AMPK, a major energy and nutrient sensor^58^, proved to follow the AGO2-distribution pattern at the arms of the midbody structure, whereas phospho-MEK, -ERK, -p38, and -JNK showed a Dicer-like-distribution motif, being mainly concentrated in the ring and at the tips of the midbody arms. Interestingly, phospho-AGO2 Ser^387^ follows the phospho-MEK/ERK/p38/JNK expression pattern, exhibiting a high concentration in the ring area. Phosphorylation of AGO2 at Ser^387^ by the activated p38 MAP Kinase is induced by cellular stress^59^. In accordance, a selective inhibitor of the p38 MAP Kinase (SB203580), causes relocalization of phospho-AGO2 Ser^387^ in a number of cytokinetic bridges, shifting its location from the ring to the midbody arms. The detection of both p38-dependent phospho-AGO2 Ser^387^ and Dicer in the midbody ring provides evidence for the existence of an active RISC machinery, suggesting a yet unrecognized role of the complex in cell division.

The dividing cells are under tremendous stress, as corroborated by our findings regarding the existence of stress-related proteins such as phospho-Akt, -AMPK, -ERK, -MEK, -JNK, and -p38-activated kinases in the midzone. In conjunction with the localization of AGO2 and phospho-AGO2, these findings underline the probable implementation of AGO2 in the regulation of spatio-temporal homeostasis in order to beneficially manage local stress. The stress-sensitive nature of AGO2 was further supported by heat-shock treatments that resulted in malformed or abolished AGO2 close-ended protrusions and by the strong colocalization pattern with the stress-related proteins Upf1 and TIAR. Moreover, the presence of phospho-eIF2*α* and ATF4 (ER-stress) proteins into the AGO2-enriched cytokinetic structures further suggests the simultaneous and synergistic activation of a MAPK-signaling network and the RNAi machinery in response to stress, in a topology-specific manner, most likely for the restoration of local transcript homeostasis during cytokinesis.

In conclusion, we herein demonstrate the AGO2 involvement in the tubular protrusions’ locasome, including open-ended tunneling nanotubes and close-ended cytokinetic bridges. Altogether, our results provide evidence for the discovery of an AGO2-niche in the midzone carrying essential properties for successful cytokinesis and cell-division integrity, although further investigation needs to be performed to thoroughly dissect the exact roles of AGOs, and their protein- and RNA-interactors, in a cell-division setting.

## Methods

### Cell culture, transfection, and treatments

NTHY ori 3-1 cell line (ECACC, Sigma-Aldrich, UK, 90011609) was cultured in RPMI 1640 growth medium (Gibco, Thermo Fisher Scientific, USA, 61870-010), while HepG2 (ATCC® HB-8065™, LGC standards, UK), LX-2 (SCC064, Sigma-Aldrich), A375 (ATCC® CRL-1619™), HCT116 (91091005, Merck, USA), HMEC (ATCC^®^ PCS-600-010™), and MDA-MB-231 (ATCC® HTB-26™) cells were maintained and grown in DMEM (41966-029, Gibco, Thermo Fisher Scientific) in standard conditions (37 °C and 5% CO_2_). The medium was supplemented with 10% fetal bovine serum (16000044, Thermo Fisher Scientific, Gibco, USA), 1% penicillin/streptomycin (10378016, Thermo Fisher Scientific, Gibco), and 1% L-glutamine (Life Technologies, USA). Cells were transfected with EGFP-hAGO2 (#21981, Addgene plasmid, UK), an AGO2-expression plasmid, using Lipofectamine 2000 (Thermo Fisher Scientific), according to the manufacturer’s instructions. For transient AGO2-expression, cells were collected and processed 24 h post transfection, for further assays. NTHY ori 3-1 cells were treated with 0.4 μg/μl Demecolcine (D7385, Sigma-Aldrich) for 3, 5, and 7 h, and 10 μΜ Cytochalasin D (C8273, Sigma-Aldrich), for 30 min. NTHY ori 3-1 cells were also exposed to 20 μΜ SB203580 (Cell Signaling Technology, Inc., USA) for 2 h. Heat-shock was performed for 16 h at 41 °C, followed by 3 h cell recovery at 37 °C. Knockdown of *AGO2* was achieved by siRNA (18 nM of siRNA-AGO2: 5’ *GUC CGU GAA UUU GGA AUC UGA CCA UGA UUC CAA AUU* 3’*)*, using the Lipofectamine 2000 -transfection- reagent (Invitrogen, USA), according to TriFECTa RNAi Kit.

### Immunostaining

Cells cultured on 10 mm round coverslips were fixed in 4% paraformaldehyde (Sigma-Aldrich, St. Louis, MO, USA) solution for 10 min at room temperature. The cells were membrane-permeabilized by incubating in 0.1% Triton-X 100 (Sigma-Aldrich, St. Louis, MO, USA), followed by blocking with 5% BSA for 1 h and exposed to the appropriate primary antibody overnight (16 h) at 4 °C. The secondary antibody solution was applied for 1 h at room temperature. All the antibodies and the concentrations applied are included in Supplementary Table S2. Vectashield^®^ Mounting Medium with DAPI (Vector Laboratories, Inc., CA 94010, USA) was added in order to visualize the nuclei at 405 nm excitation wavelength.

### In situ proximity ligation assay (iPLA)

Cells were cultured on coverslips, rinsed with DUO link wash Buffer B according to the manufacturer’s protocol (DUO92007, Sigma-Aldrich, USA). Then they were incubated in blocking solution for 30 min at 37 °C, and the appropriate primary antibody was applied overnight at 4 °C. Next, cells were exposed to secondary antibodies conjugated to oligo-nucleotide iPLA probes (cat DUO92001, cat DUO92005, MINUS and PLUS, Sigma-Aldrich) for 1 h at 37 °C. Ligase activity performed coupled with rolling circle amplification and the products were hybridized by oligo-nucleotide probes labeled with a fluorophore. Red punctuate signals were captured using confocal scanning microscopy.

### Quantitative PCR analysis

Total RNA extract from NTHY ori 3-1 cells was isolated using Trizol according to the manufacturer’s protocol. Next, cDNA was synthesized using as a template ∼500 ng of total RNA and the Quantitect RT kit, Qiagen, 205311Q according to the manufacturer’s recommendations. The cDNA was amplified in triplicates on a Roche Light Cycler 96 System. Relative gene expression was calculated using the 2^−ΔΔCt^ method. Expression of genes of interest (goi) was normalized by the reference gene *Lamin*. Fold change in gene expression was calculated as 2^−ΔΔCT ^^60^, where ΔΔC_T =_ ΔC_T(goi)_ − ΔC_T(Lamin)_. The relative change of *Ago2* following the treatment with siAgo2 was calculated using the gene expression of the untreated scrambled control cells as a control.

### Chromosome measurements

Chromosome preparations were obtained from logarithmically growing HCT116 cell cultures after been exposed to Colcemid (0.1 µg/ml) (Gibco, Grand Island, USA) for 1–3 h, at 37 °C in 5% CO_2_. Cells were harvested by trypsinization (Gibco, Grand Island, USA), exposed in hypotonic solution (5 ml of 0.075 M KCl, Sigma), and fixed [3× methanol (Applichem GmbH, Darmstadt, Germany)/1× CH3COOH (Merck, Darmstadt, Germany)]. For karyotypic analysis^61^, we applied inverted DAPI banding: slides were stained and mounted with 0.6 μg/ml DAPI in Vectashield antifade medium (Vector Laboratories, USA). Cytogenetic analyses were performed using a 63× magnification lens on a fluorescent Axio-Imager Z1, Zeiss microscope, equipped with a MetaSystems charge-coupled device (CCD) camera, and the MetaSystems Isis software (MetaSystems GmbH, Germany). Karyotypes were recorded according to International System for Human Cytogenetic Nomenclature 2016. Micronuclei analysis was performed in cytologic preparations treated as above and stained by DAPI.

### Microscopy

Images were acquired with a Leica TCS SP5 inverted CLSM, using Leica HC PLAPO 63×1.4NA CS immersion objectives. Acquisition was performed sequentially to avoid cross-talk. Time-lapse videos were started 18 h post transfection at 30 sec or 1 min time-lapse intervals for 45 min.

For TEM, the cells were cultured in 35 mm CellStar cell culture dishes (Greiner Bio-One, GmbH, Germany), on a clarfilm, for 24–48 h, under standard conditions, fixed in 2.5% glutaraldehyde (in 0.1 M phosphate buffer), and post-fixed with 1% osmium tetroxide. Next, samples were dehydrated via a graded series of ethanol concentrations, 30, 50, 70, 90, and 100%, followed by propylene-oxide (PO) treatment. They were further processed in a mixture of Epon/Araldite resins diluted in PO, flat-embedded in fresh epoxy-resin mixture, and allowed to polymerize at 60 °C for 24 h. Small epoxy pieces were peeled away from petri-dishes, glued on epoxy blocks, and allowed to polymerize for 24 h. Ultra-thin sections of 65–70 nm (Leica EM UC7 Ultra-microtome, Leica Microsystems, Austria) were mounted onto 200 mesh nickel grids, stained with uranyl acetate and lead citrate, examined with a Philips 420 transmission electron microscope at an acceleration voltage of 60 kV, and photographed with a MegaView G2CCD camera (Olympus SIS, Germany).

SEM was performed with a Jeol 6380LV electron microscope (JEOL Ltd., USA) that was operated at 15 kV and was equipped with an Oxford EDS system for chemical analysis. The XRD patterns were recorded by means of a Siemens D5005 (Bruker AXS, USA) using Cu K radiation. For the evaluation of the surface structural configuration, NTHY ori 3-1 cells were prepared and cleansed with ethanol. For the electron microscopy imaging and analysis, cells were attached on aluminum stubs and were analyzed in a low-vacuum mode (around 30 Pa) to minimize cell stress due to the progressive lack of humidity of the samples during the process in instrument’s vacuum chamber, as well as to eliminate charging effects after the cells have dried.

Details for the materials, reagents, drug compounds, and antibodies (primary and secondary) used in the described Methods are presented in Supplementary Table S1.

### Protrusion measurements

The number and length of filopodial protrusions were measured in 102 cells from CLSM fluorescence images (three independent biological experiments) (Supplementary Fig. S3a, b) using the FiloDetect algorithm^62^. The algorithm is implemented in Matlab and was suitably modified so that filopodia shape could also be modeled. A filopodium’s midpoint width was defined as the number of pixels occupied on the minor axis of the ellipse created by Matlab’s “regionprops” function. The corresponding numbers for parallel translations of the minor axis for a distance equal to 2/3 of half the major axis toward both ends of the major axis were also evaluated. Between these two points the one at greater distance from the cell’s centroid (also identified using “regionprops”) was defined to be the filopodium tip width. The shape model was obtained by evaluating the average tip-to-midpoint width ratio among all filopodia detected in the cells measured. The same procedure was followed for the calculation of the corresponding quantities for AGO2 protrusions in 66 cells. Since this type of protrusion is unique per cell no averages were required. The AGO2 protrusion of each cell along with other protrusion-like structures was automatically identified using Matlab’s “fibermetric” function. To isolate the real AGO2 protrusion among them, a region of interest was manually cropped around it and superimposed with the “fibermetric” image.

### Image analysis

3D surface reconstruction was implemented using Imaris 9.1.2 (Bitplane, South Windsor, CT, USA). Images used for colocalization analysis were acquired with Nyquist sampling; the Richardson-Lucy Total Variation algorithm^63^ was selected from DeconvolutionLab2 plugin^64^ for image deconvolution. Theoretical PSFs according to the Born and Wolf 3D Optical Model were constructed from the PSF Generator plugin^65^. Different channels were processed independently. Further preprocessing consisted of background subtraction (3D rolling ball algorithm^66^) and thresholding (triangle method^67^) all run in Fiji^68^. The Pearson correlation coefficient (*R*), and Manders’ coefficients (*M*1, *M*2) were used to quantify colocalization. *R* measures the correlation between probes, while *M*1 and *M*2 are measures of co-occurrence; *M*1 captures the proportion of above-threshold pixels in the red channel occupying above-threshold pixels of the green channel, and vice versa for *M*2. Controls for specificity of primary and secondary antibodies (Supplementary Fig. S9c, g, h), autofluorescence (Supplementary Fig. S9d), specificity of PLA interaction foci (Supplementary Fig. S9e), colocalization analysis (Supplementary Fig. S9f), negative control (scramble) of siAGO2 (Supplementary Fig. S3a), and biological colocalization negative control (Supplementary Fig. S3b) are provided.

### Statistics and reproducibility

One-tailed Student’s *t*-test^69^ was used to evaluate the significance of Pearson (*R*) and Manders’ values. The sample size for each test was *n* = 6 fluorescence images and the *α*-level was set to 0.01. Mann–Whitney *U*-tests (two-sided) were used to compare the differences obtained in the lengths and ratios between Actin-filopodial and AGO2 protrusions. Although filopodial protrusions are log-normally distributed^25^, a non-parametric test was chosen since q-q plot and Shapiro test did not reveal a similar distribution for AGO2 protrusions. Actin-filopodial protrusions were measured on 102 cells (at least *n* = 3 biologically independent experiments, statistical sample size *n* = 3849 Actin-filopodial protrusions) while AGO2 protrusions on 66 cells (*n* = 5 biologically independent experiments, statistical sample size *n* = 66 AGO2 protrusions). The *α*-level was set at 0.01 for both tests. *z*-test was employed to test the proportion of non-lagging chromosomes (*n*1 = 176 GFP cells, *n*2 = 169 AGO2-GFP cells), micronuclei formation (*n*1 = 174 GFP cells, *n*2 = 168 AGO2-GFP cells), and chromosomal deregulation (*n*1 = 188 GFP cells, *n*2 = 182 AGO2-GFP cells) in GFP/AGO2-GFP assays. The same procedure was employed to test the proportion of micronuclei formation (*n*1 = 117 SCR cells, *n*2 = 117 siAGO2 cells), and chromosomal deregulation (*n*1 = 103 SCR cells, *n*2 = 114 siAGO2 cells) in SCR/siAGO2 knocked down cells. For the analyses three biologically independent experiments were conducted. The use of *z*-test was justified based on the expected frequencies that occurred in all our measurements^70,71^. The *α*-level was set at 0.01.

### Reporting summary

Further information on research design is available in the Nature Research Reporting Summary linked to this article.

## Supplementary information

Supplementary Information

Description of Additional Supplementary Files

Supplementary Movie 1

Supplementary Movie 2

Supplementary Movie 3

Supplementary Movie 4

Reporting Summary

## Data Availability

All raw images along with the corresponding processed ones can be found at Zenodo (10.5281/zenodo.4415734). All other data are available from the corresponding author on reasonable request.

## Code availability

All scripts used can be found at Zenodo (10.5281/zenodo.4415734). Imaris 9.1.2, Matlab 2018a, and R version 4.0.3 were used. Further details on the procedure of measurements and corresponding statistical tests for all relevant figures can be found in the “Notes.txt” file at the Online Repository.
