# Supplementary material for: AGO2 localizes to cytokinetic protrusions in a p38-dependent manner and is needed for accurate cell division
Source: Commun Biol. 2021 Jun 11;4:726. doi: 10.1038/s42003-021-02130-0 (PMC8196063; doi:10.1038/s42003-021-02130-0)
Supplement: Supplementary file 1 — Supplementary Information [file 42003_2021_2130_MOESM1_ESM.pdf]

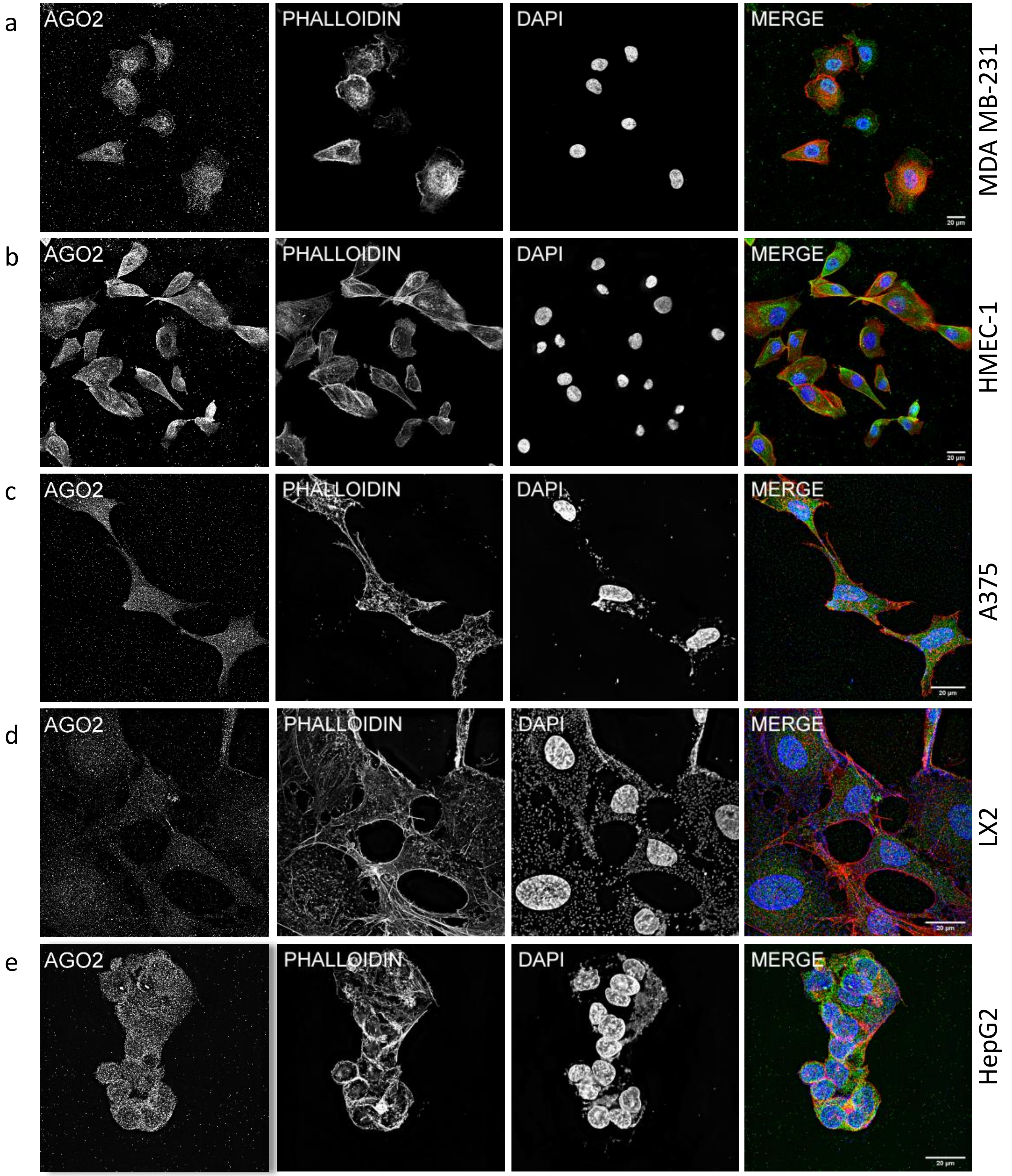

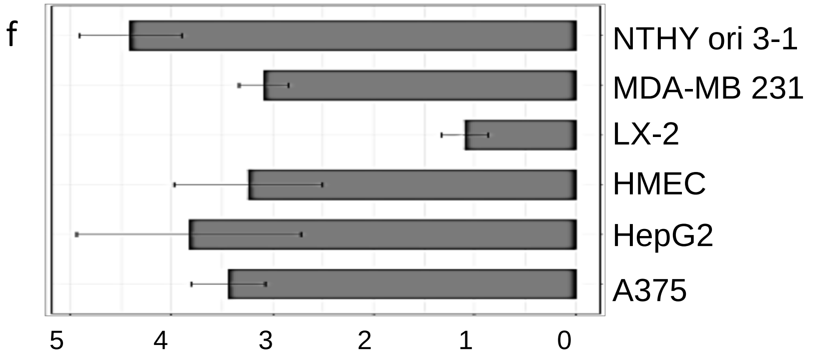

Figure S1: Subcellular distribution of AGO2 in different cell lines. Representative images of (a) MDA MB-231, (b) HMEC, (c) A375, (d) LX-2 and (e) HepG2 cells. AGO2 in green, F-Actin (phalloidin) in red and nuclei in blue (DAPI) (scale bar: 20µm). (f) Barplot of AGO2 mean signal intensity per cell in the respective cell lines measured by Imaris using 3D surface reconstruction of confocal images.

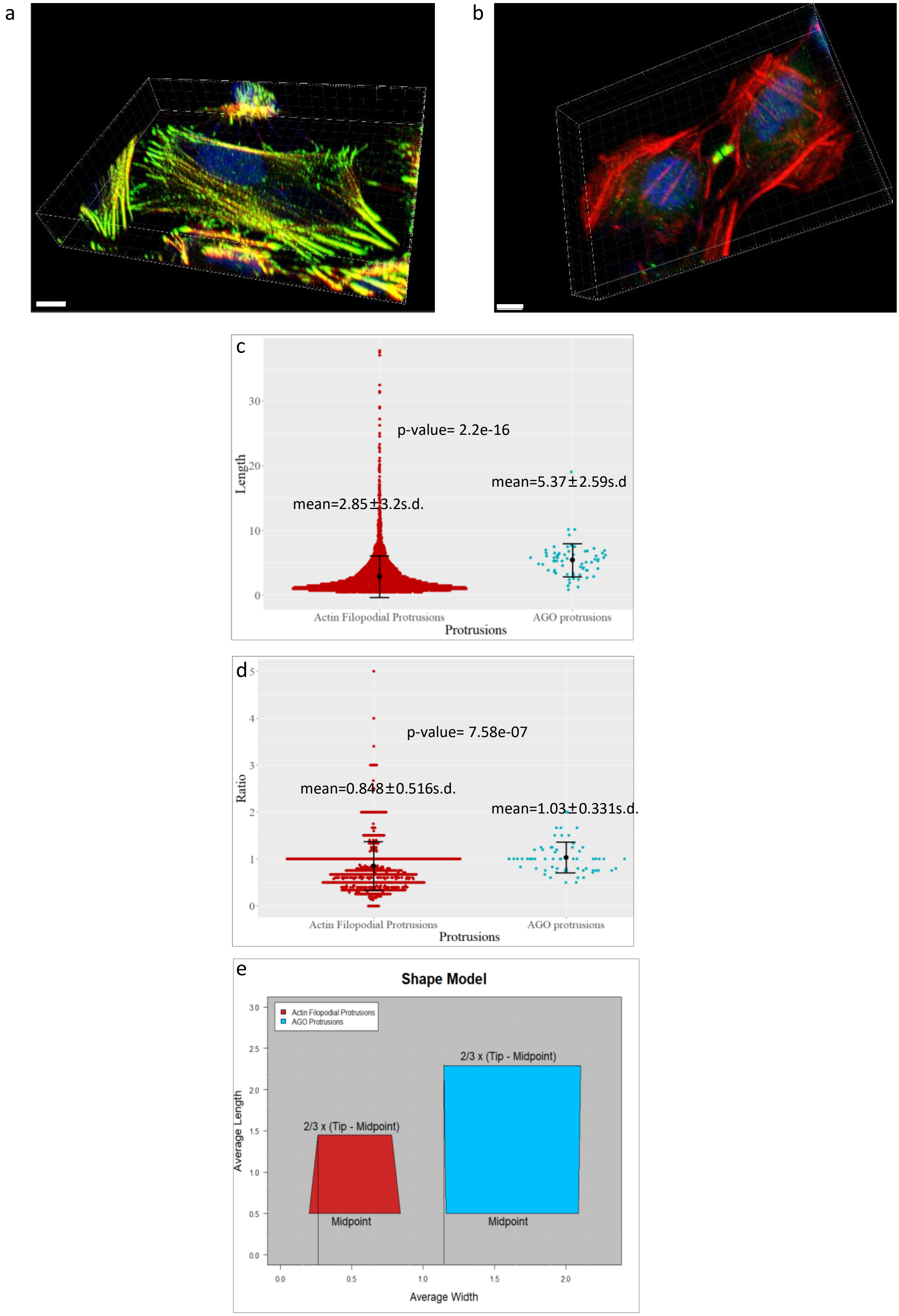

Figure S2: AGO2 close-ended structures differ from the typical Actin-filopodial protrusions. 3D signal reconstruction of representative images of NTHY ori 3-1 cells. (a) F-Actin filopodial (Actinin 4, green; F-Actin [Texas red-phalloidin], red, scale bar: 10 μm) and (b) AGO2 protrusions (AGO2 in green; F-Actin [phalloidin] in red, respectively, scale bar: 8 μm). Sina-plot of Actin-filopodial (at least n=3 biologically independent experiments, statistical test sample size n=3849 filopodial protrusions) and AGO2 protrusion (at least n=5 biologically independent experiments, statistical test sample size n=66 AGO2 protrusions) (c) lengths and (d) tip-to-midpoint width ratios. (e) Shape model based on protrusion mean value of tip-to-midpoint width ratios.

**Pairs for lineplots**		**Pearson**	**Manders’**	
		R	M1	M2
AGO2 - CITK	mean	0.126	0.221	0.336
	p-value	0.0007	0.0002	0.0007
AGO2 - AURORA B	mean	0.718	0.889	0.786
	p-value	8.00e-07	6.68e-07	4.62e-07
AURORA B - CITK	mean	0.172	0.323	0.461
	p-value	8.19e-06	9.43e-06	2.73e-05
AGO2 - DICER	mean	0.221	0.350	0.719
	p-value	0.0017	0.0007	0.0004
AURORA B - DICER	mean	0.233	0.342	0.593
	p-value	0.0181	0.0008	0.0004
AGO2 - αTUB	mean	0.770	0.786	0.958
	p-value	4.1e-08	1.36e-07	1.28e-09
AGO2 - PHALLOIDIN	mean	0.153	0.328	0.203
	p-value	0.0087	0.0099	0.0068
αTUB - PHALLOIDIN	mean	0.266	0.368	0.602
	p-value	0.0138	0.0096	0.0004
αTUB - DICER	mean	0.261	0.387	0.815
	p-value	0.0012	0.0006	3.15e-06
DICER - PHALLOIDIN	mean	0.077	0.291	0.148
	p-value	0.0088	0.0102	0.0306
PHALLOIDIN - LAMIN A/C (non-colocalized area)	mean	0.024	0.003	0.340
	p-value	0.0916	0.1257	0.0263

Table S1: Colocalization metrics (Pearson [R] and Manders’ [M1 and M2] coefficients) and respective p-values of the paired proteins depicted in yellow ROIs in Fig. 3e-h, Fig. 4c-e, Fig. S4a-c.

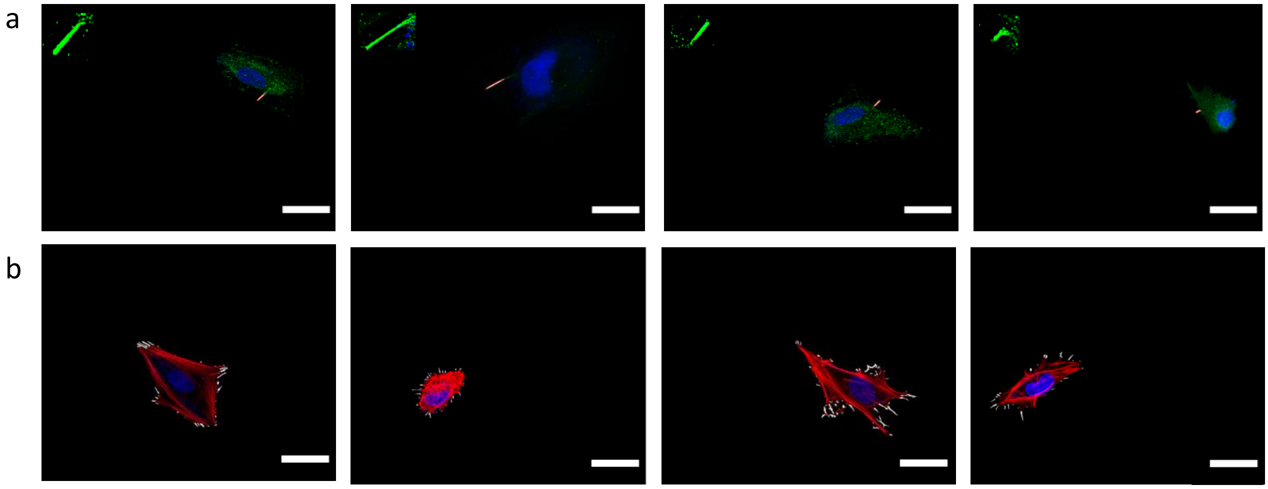

Figure S3: Examples of the output of the Matlab script used to detect, measure and yield statistics shown in Figure S2 from the comparative analysis of (a) AGO2 and (b) Actin-Filopodial protrusions. The identified protrusions are colored white. All measurements were made according to the ellipses created by Matlab's “regionprops” function [shown in red in (a)]. In the top-left corner of (a) the manually cropped protrusions used to extract the AGO2 protrusion among those identified by Matlab's “fibermetric” function are overlaid. Scale bar: 40μm.

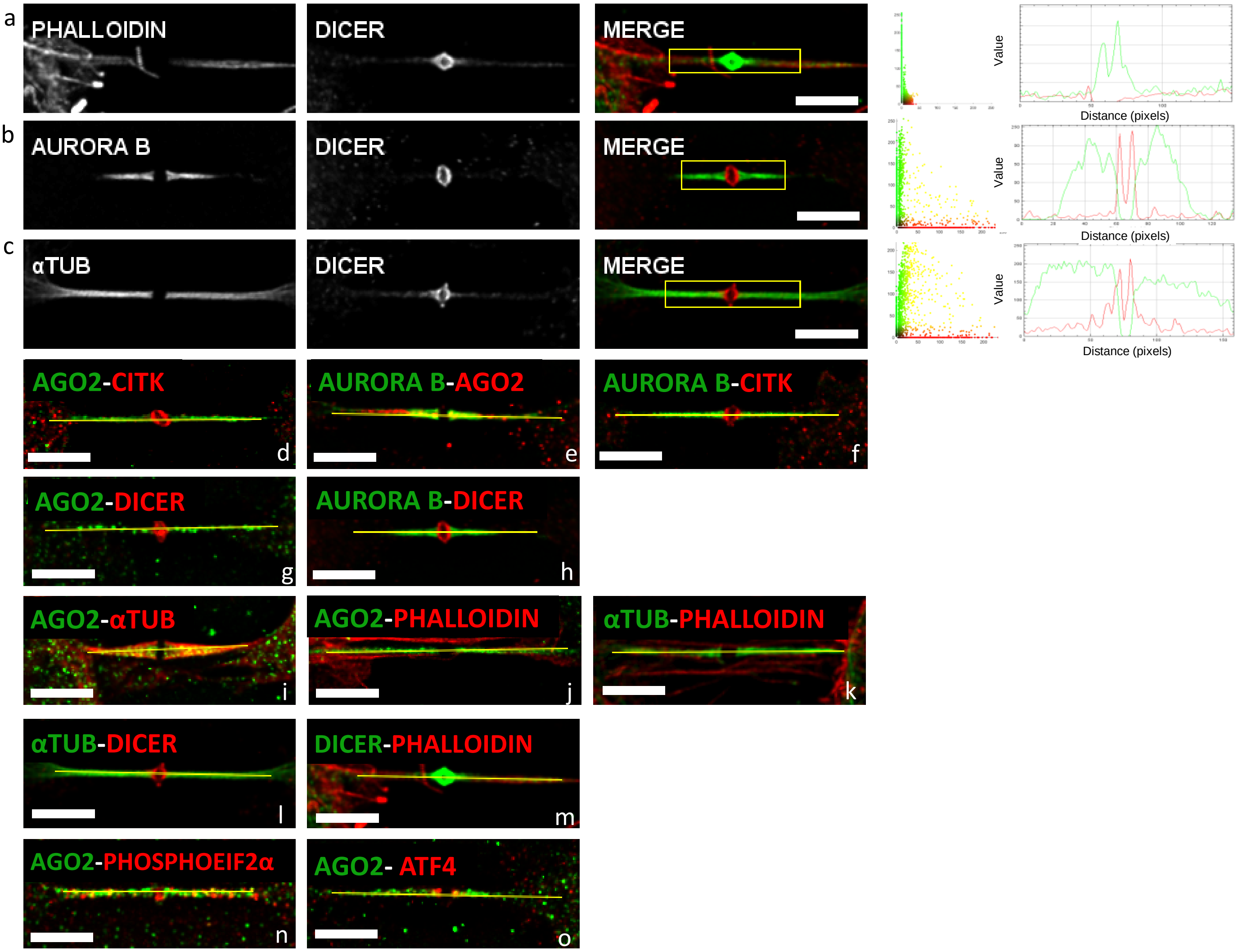

Figure S4: Colocalization analysis of the intercellular bridge content. (a-c) Representative images of NTHY ori 3-1 cells and visual assessment (merged images, scatterplots and intensity lineplots) of colocalization. The colocalization metrics (Pearson [R] and Manders' [M1 and M2] coefficients) and respective p-values are presented in Table S2 (n=6 biologically independent samples). (d-o) Lineplots indicate the signal intensity of Dicer and the paired protein along the lines. Line overlays on merged channels from all conditions shown in Fig. 3e-h, Fig. 4c-e, Fig. 10e-f and Fig. S4a-c used to obtain the corresponding lineplots. Scale bar: 5μm.

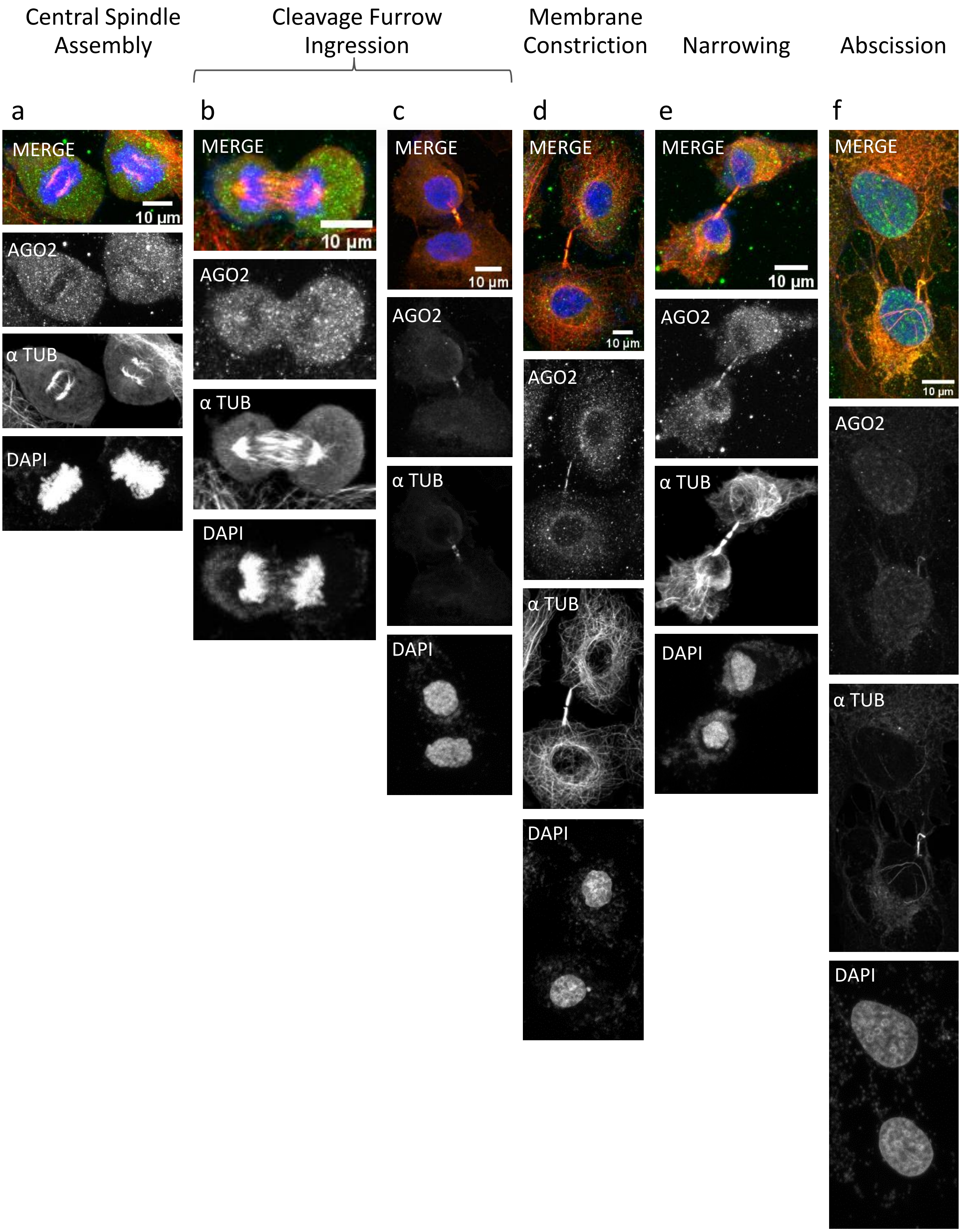

Figure S5: AGO2 distribution during cell division. Representative immunofluorescence images of NTHY ori 3-1 cells. AGO2 colocalization with *α*-Tubulin was visualized in a time-course manner. AGO2 in green. *α*-Tubulin in red and nuclei in blue (DAPI). Images depict (a) central spindle, (b,c) assembly cleavage furrow ingression, (d) membrane constriction, (e) narrowing and (f) abscission (scale bar: 10 µm).

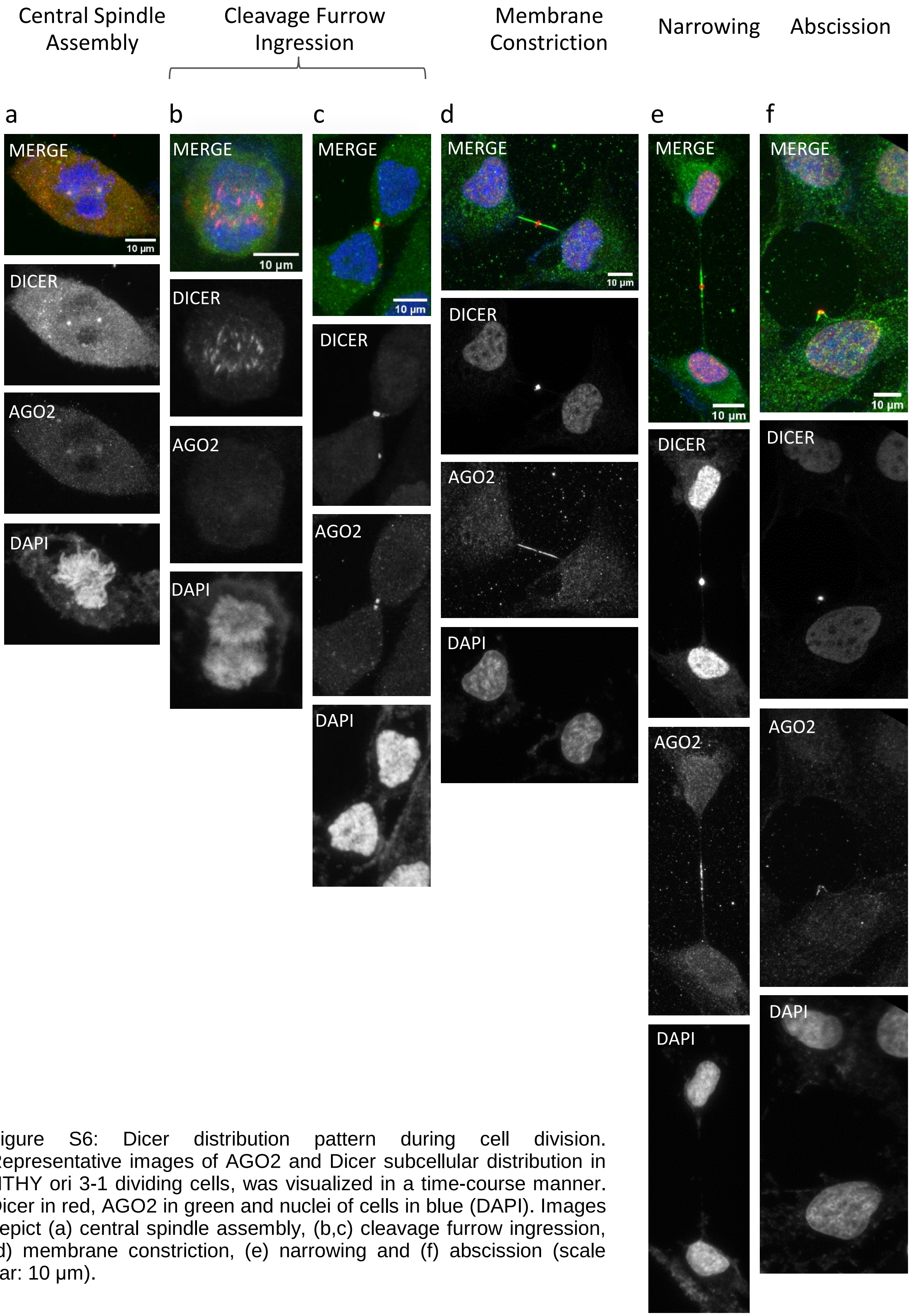

Figure S6: Dicer distribution pattern during cell division. Representative images of AGO2 and Dicer subcellular distribution in NTHY ori 3-1 dividing cells, was visualized in a time-course manner. Dicer in red, AGO2 in green and nuclei of cells in blue (DAPI). Images depict (a) central spindle assembly, (b,c) cleavage furrow ingression, (d) membrane constriction, (e) narrowing and (f) abscission (scale bar: 10 µm).

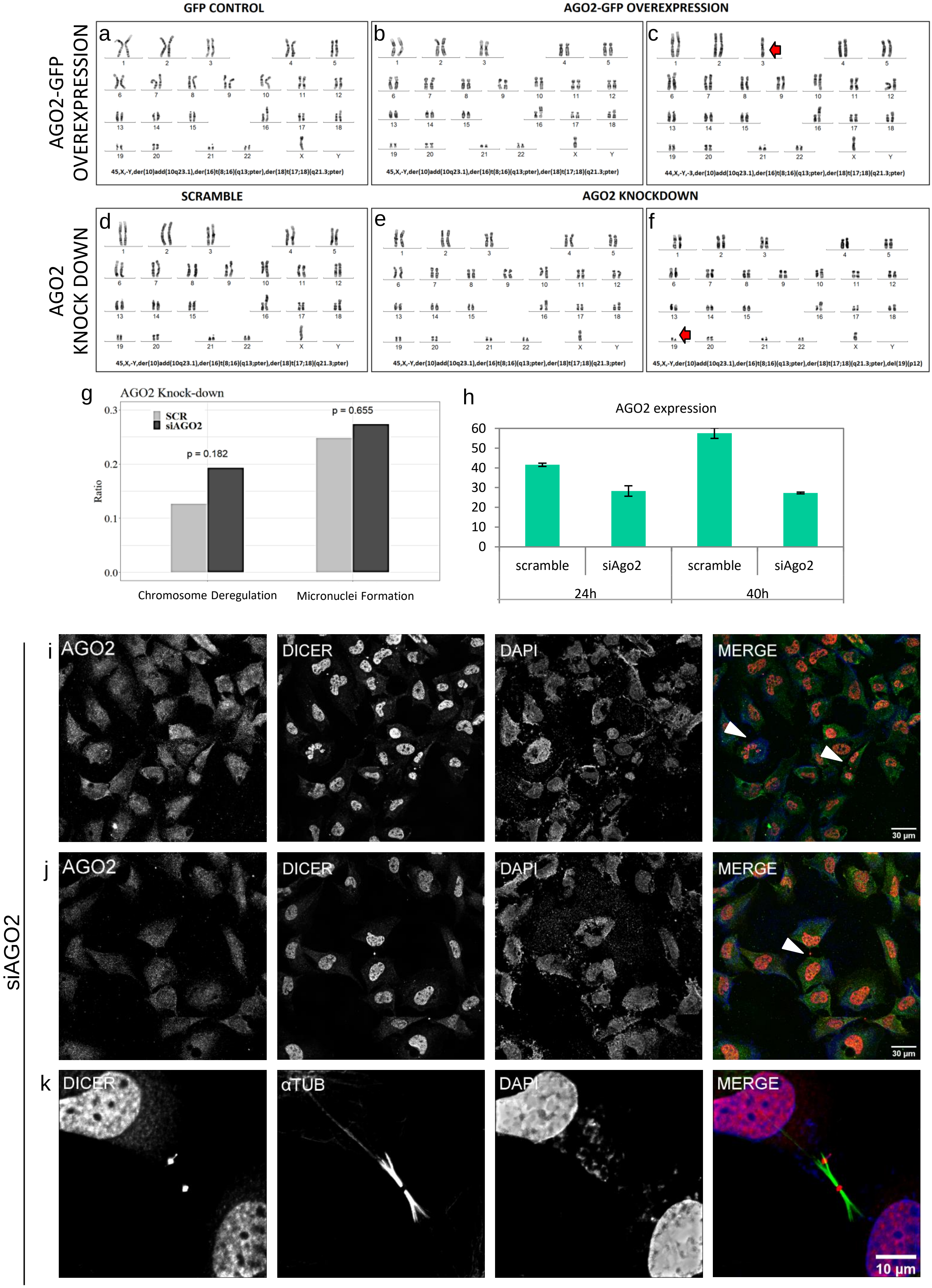
GFP CONTROL
AGO2-GFP OVEREXPRESSION
AGO2-GFP OVEREXPRESSION
a
b
c
45,X,-Y,der(10)add(10q23.1),der(16)t(8;16)(q13;pter),der(18)t(17;18)(q21.3;pter)
45,X,-Y,der(10)add(10q23.1),der(16)t(8;16)(q13;pter),der(18)t(17;18)(q21.3;pter)
44,X,-Y,-3,der(10)add(10q23.1),der(16)t(8;16)(q13;pter),der(18)t(17;18)(q21.3;pter)
SCRAMBLE
AGO2 KNOCKDOWN
AGO2 KNOCK DOWN
d
e
f
45,X,-Y,der(10)add(10q23.1),der(16)t(8;16)(q13;pter),der(18)t(17;18)(q21.3;pter)
45,X,-Y,der(10)add(10q23.1),der(16)t(8;16)(q13;pter),der(18)t(17;18)(q21.3;pter)
45,X,-Y,der(10)add(10q23.1),der(16)t(8;16)(q13;pter),der(18)t(17;18)(q21.3;pter),del(19)(p12)
g
AGO2 Knock-down
SCR
siAGO2
p = 0.182
p = 0.655
Ratio
Chromosome Deregulation
Micronuclei Formation
h
AGO2 expression
scramble
siAgo2
scramble
siAgo2
24h
40h
siAGO2
i
j
k
AGO2
DICER
DAPI
MERGE
αTUB
30 µm
10 µm

Figure S7: AGO2 over-expression and down-regulation effect on cell division. Karyotypes of HCT116 (a) GFP-control cells and (b,c) AGO2-GFP over-expressing cells. Karyotypes of HCT116 (d) cells transfected with scramble control and (e,f) AGO2-knocked down cells. (g) Bar plot of the effects of AGO2 down-regulation in HCT116 cells on numerical chromosome instability and micronuclei formation. z-test was used to evaluate their significance. Bar height indicates the ratio of deregulated chromosomes (n=2 biologically independent experiments, statistical sample size n1=103 SCR cells, n2=114 siAGO2 cells) and cells with micronuclei formation (n=2 biologically independent experiments, statistical sample size n1=117 SCR cells, n2=117 siAGO2 cells), respectively (h) Bar plot indicates AGO2 expression levels in 24 and 40h following siAGO2 transfection determined by quantitative PCR analysis. (i-k) Representative images of NTHY ori 3-1 AGO2 knocked-down cells. White arrowheads indicate abnormalities such as (i) abnormal nucleus (scale bar: 30 μm), (i,j) malformed intercellular bridges (scale bar: 30 μm) and (k) double midbody ring (scale bar: 10 μm). (i,j) AGO2 and (k) *α*-Tubulin in green and (i-k) Dicer in red. Nuclei in blue (DAPI).

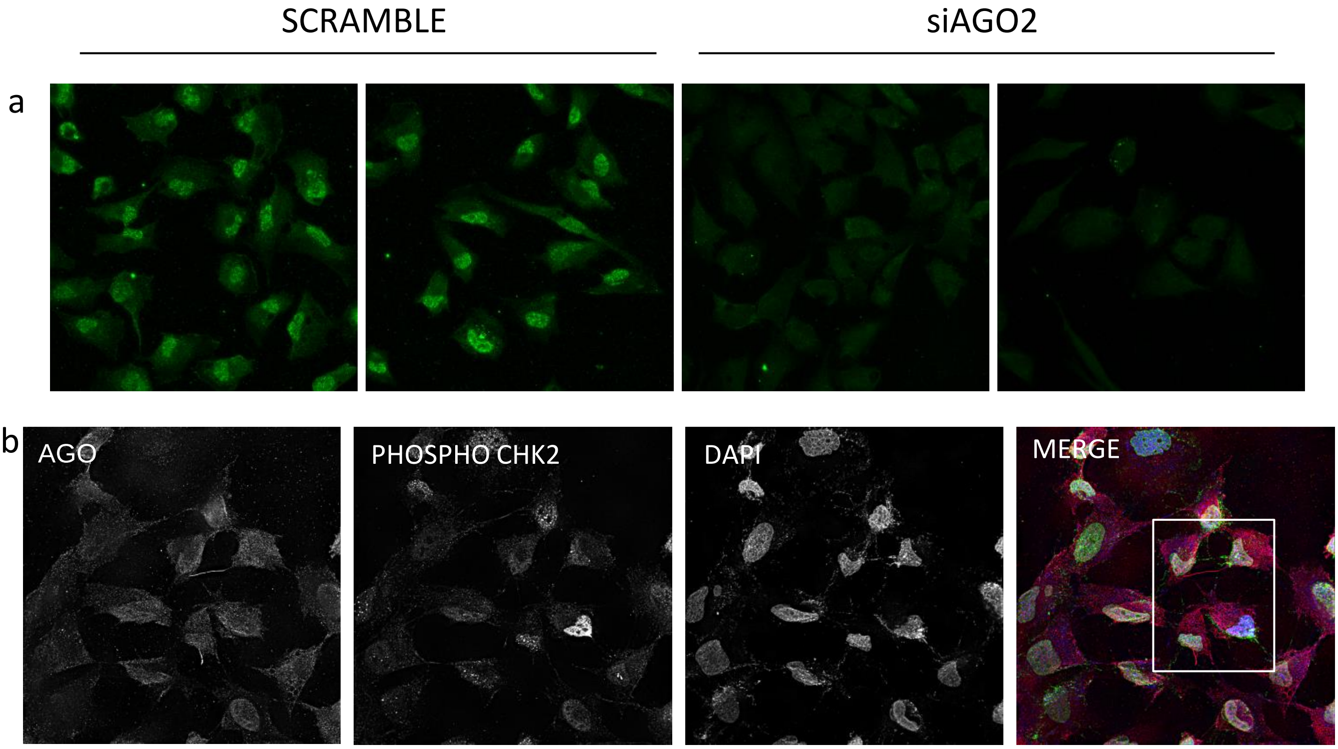

Figure S8: Negative controls. Representative images of NTHY ori 3-1 cells. Lack of colocalization distribution pattern of AGO2-phospho CHK2 across the intercellular bridge. (a) Differential signal intensities of AGO2 in siAGO2 cells compared to the scrambled ones, (b) AGO2 (green), phospho-CHK2 (red), nuclei (blue).

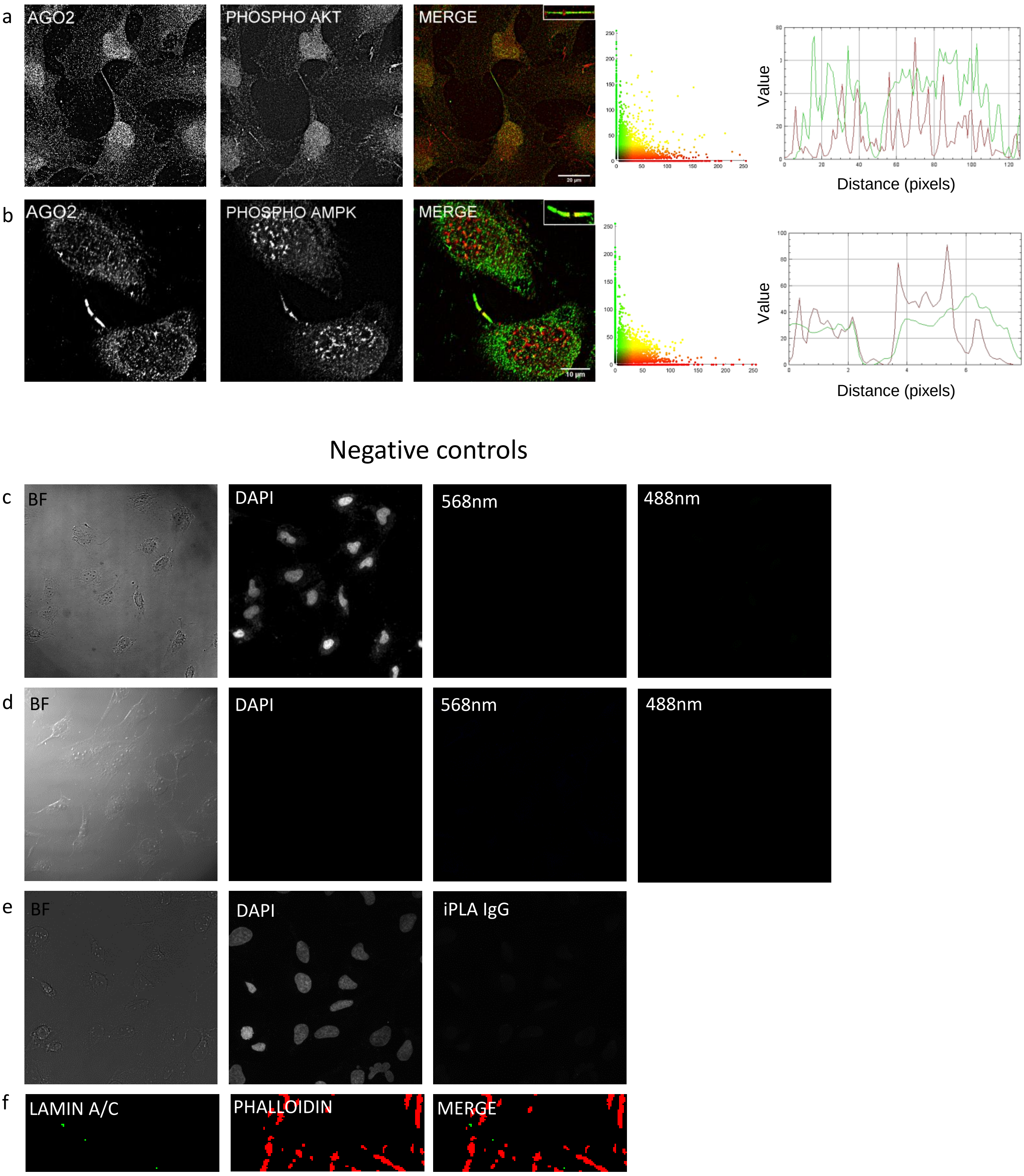
a
AGO2
PHOSPHO AKT
MERGE
Value
Distance (pixels)
b
AGO2
PHOSPHO AMPK
MERGE
Value
Distance (pixels)
Negative controls
c
BF
DAPI
568nm
488nm
d
BF
DAPI
568nm
488nm
e
BF
DAPI
iPLA IgG
f
LAMIN A/C
PHALLOIDIN
MERGE

Figure S9

Negative controls

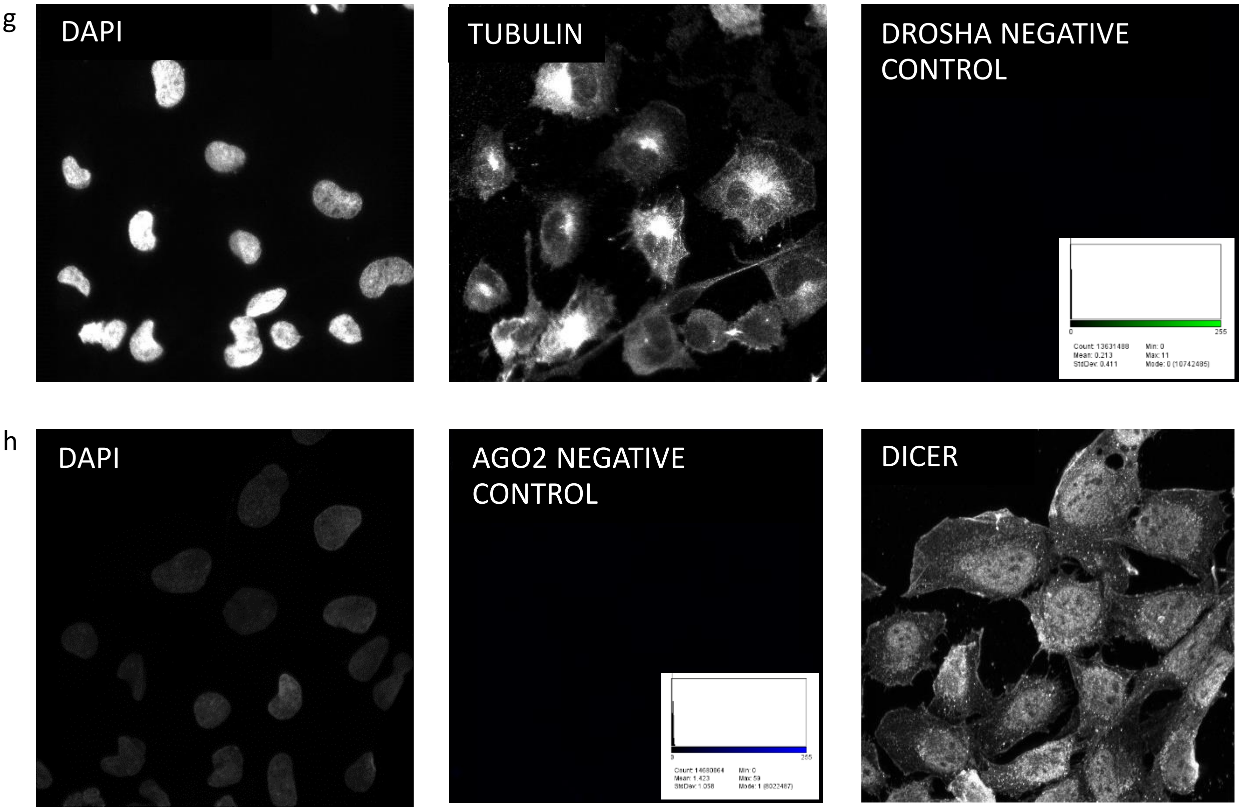

Figure S9: Phospho-Akt and phospho-AMPK kinase profiling follow the AGO2 distribution in the midbody structure. Representative images of NTHY ori 3-1 cells. (a) AGO2 (green) and phospho-Akt (red) (scale bar: 20 µm), (b) AGO2 (green) and phospho-AMPK (red) (scale bar: 10 µm). Nuclei in blue (DAPI). Visual assessment (merged images, scatterplots and intensity lineplots) of colocalization. Lineplots indicate the signal intensity of the two paired proteins. (c) Negative control of NTHY ori 3-1 cells stained with DAPI and secondary antibodies (without primary antibodies), (d) Negative control for autofluorescence (only fixed cells), (e) Negative control for PLAi experiment (IgG control), (f) Colocalization control, cytoplasmic region where Lamin A/C (green) and phalloidin (Texas-red) are known to anti- colocalize. (g) Negative control for red secondary antibody, α-Tubulin (green) and nuclei (blue). In the red channel anti-Drosha primary antibody staining without red secondary antibody. (h) Negative control for green secondary antibody, Dicer stained red and nuclei (blue) by DAPI. In the green channel anti-AGO2 primary antibody staining without green secondary antibody. (g,h) Signal histograms in the negative channels.

Continued
Figure S9

Antibodies, Chemicals, Drugs	Company	Code	Host	Concentrations
16% paraformaldehyde	Thermoscientific	28906		
Triton-X 100	Sigma/Aldrich, St. Louis, MO, USA	11332481001		
Bovine Serum Albumin	Sigma/Aldrich, St. Louis, MO, USA	B2064		
anti-α actinin 4	Abcam, Cambridge, MA, USA	ab198608	rabbit	(1/50)
anti-argonaute-2	Abcam, Cambridge, MA, USA	ab57113	mouse	(1/80)
anti-α-tubulin	Abcam, Cambridge, MA, USA	ab18251	rabbit	(1/1000)
anti-α-tubulin	Cell Signaling	DM1A#3873s	mouse	(1/100)
anti-Citron kinase	Novus	NBP2-38592	rabbit	(1/100)
anti-Argonaute-2	Abcam, Cambridge, MA, USA	ab186733	rabbit	(1/50)
anti-Drosha	Abcam, Cambridge, MA, USA	ab12286	rabbit	(1/100)
anti-DGCR8	Novus	NBP1-30115	rabbit	(1/100)
anti-Dicer	Novus	NBP1-06520	rabbit	(1/120)
anti-ATF4	GenScript	Ab-245	rabbit	(1/100)
anti-Aurora B	BD Biosciences	611082	mouse	(1/200)
anti-Staufen/STAU1	Abcam, Cambridge, MA, USA	ab50914	rabbit	(1/100)
anti-Phospho-p38 MAPK (Thr180/Tyr182)	Cell Signaling	9211	rabbit	(1/100)
anti-phospho-AMPK1 (Ser485)/AMPK 2 (Ser491)	Cell Signaling	4185	rabbit	(1/100)
anti-Phospho-MEK1/2 (Ser217/221) (41G9)	Cell Signaling	9154	rabbit	(1/100)
anti-Phospho-p38 MAPK (Thr180/Tyr182)	Cell Signaling	9211	rabbit	(1/100)
anti-Phospho-Akt (Thr308)	Cell Signaling	9275	rabbit	(1/100)
anti-Phospho-sAPK/JNK (Thr183/Tyr185)	Cell Signaling	9251	rabbit	(1/100)
Donkey Anti-Rabbit IgG (H + L) Secondary Antibody Fluor®488 conjugate	Invitrogen, Waltham, MA, USA			(1/250)
Goat anti-Rabbit IgG (H + L) Secondary Antibody Alexa Fluor 594	Invitrogen, Waltham, MA, USA			(1/250)
Goat anti-Mouse IgG (H + L) Cross-Adsorbed Secondary Antibody Alexa Fluor 488	Invitrogen, Waltham, MA, USA			(1/250)
Goat Anti-mouse IgG Dylight 594 Conjugated	Invitrogen, Waltham, MA, USA			(1/250)
Rhodamine - Phalloidin	Biotium			(5/200)
VECTASHIELD® Mounting Medium with DAPI	Vector Laboratories, Inc., 30 Ingold Road, Burlingame, CA 94010 USA			
phospho-ago kit[Argonaute 2 (a.a. 389-398), Argonaute 2 (Tyr-393) Argonaute 2 (Ser-387)]	ECM BIOSCIENCES	AK6970	rabbit	(1/50)
Upf1 antibody (D15G6)	Cell Signaling	12040	rabbit	(1/100)
TIAR antibody	Cell Signaling	8509	rabbit	(1/100)
Dynactin antibody	Cell Signaling	69399	rabbit	(1/100)
lipofectamine 2000	invitrogen	11668-027		
CF 488 anti-streptavidin	Biotium	#29034		(1/250)
biotinylated oligo(dT) probe	biotrans	Z5261		(1/50)
DsiRNAs and TriFECTa Kits in tubes	IDT			according to the manufacturer's protocol
Phospho-eIF2α (Ser51) (D9G8)	Cell Signaling	3398	rabbit	(1/100)
Duolink™ proximity ligation assay (PLA)	Merck	92101		according to the manufacturer's protocol
anti-phospho-p44/42 MAPK (Erk1/2) (Thr202/Tyr204)	Cell Signaling	9101	rabbit	(1/100)
demecolcine	Sigma/Aldrich, St. Louis, MO, USA	D7385		0,4µg/ml
cytochalasin D	Sigma/Aldrich, St. Louis, MO, USA	C8273		10µM
anti-chk2 (1C12)	Cell Signaling	3440	mouse	(1/100)
anti-chk1	Cell Signaling	2345	rabbit	(1/100)
anti-phospho-Chk1 (Ser345)	Cell Signaling	2341	rabbit	(1/100)
anti-phospho-Chk2 (Thr68)	Cell Signaling	2661	rabbit	(1/100)
anti-lamina/c	Abcam, Cambridge, MA, USA	ab108595	rabbit	(1/400)
anti-Wee1	Cell Signaling	4936	rabbit	(1/100)
anti phospho Wee1 (Ser642) (D47G5)	Cell Signaling	4910	rabbit	(1/100)
anti-phosphop53	Cell Signaling	9286	mouse	(1/100)
p53	Cell Signaling	2433	rabbit	(1/100)
TRI Reagent	Sigma/Aldrich	T9424		

Table S2: Reagents, drug compounds and antibodies (primary and secondary). Details of the materials used in the methodology of the manuscript.
