# Supplementary material for: AGO2 localizes to cytokinetic protrusions in a p38-dependent manner and is needed for accurate cell division
Source: Commun Biol. 2021 Jun 11;4:726. doi: 10.1038/s42003-021-02130-0 (PMC8196063; doi:10.1038/s42003-021-02130-0)
Supplement: Supplementary file 2 — Description of Additional Supplementary Files [file 42003_2021_2130_MOESM2_ESM.pdf]

Description of Additional Supplementary Files

File name: Supplementary movie 1
Description: AGO2-GFP distribution in open-ended tubes. Time-lapse video depicting the motion of AGO2-GFP in open-ended tubes. The time-lapse video was started 18 hours post- AGO2-GFP-transfection and were captured at 30 seconds time-lapse intervals for 45 minutes.

File name: Supplementary movie 2
Description: AGO2-GFP distribution in open-ended tubes. Time-lapse video depicting the motion of AGO2-GFP in open-ended tubes. The time-lapse video was started 18 hours post-AGO2-GFP-transfection and were captured at 20 seconds time-lapse intervals for 45 minutes.

File name: Supplementary movie 3
Description: AGO2-GFP distribution in cytokinetic structure of close-ended tubes. Time-lapse video monitoring AGO2-GFP motion throughout the cytokinetic intercellular bridge. The time-lapse video was started 18 hours post- AGO2-GFP-transfection and were captured at 30 seconds time-lapse intervals for 45 minutes.

File name: Supplementary movie 4
Description: AGO2-GFP distribution in cytokinetic structure of close-ended tubes. Time-lapse video monitoring AGO2-GFP motion throughout the cytokinetic intercellular bridge. The time-lapse video was started 18 hours post- AGO2-GFP-transfection and were captured at 30 seconds time-lapse intervals for 45 minutes. The video is processed using the “Find Edges” algorithm of Image J processing tool box

.
