# Supplementary material for: AGO2 localizes to cytokinetic protrusions in a p38-dependent manner and is needed for accurate cell division
Source: Commun Biol. 2021 Jun 11;4:726. doi: 10.1038/s42003-021-02130-0 (PMC8196063; doi:10.1038/s42003-021-02130-0)
Supplement: Supplementary file 7 — Reporting Summary [file 42003_2021_2130_MOESM7_ESM.pdf]

nature research

Corresponding author(s): Ema Anastasiadou, COMMSBIO-19-1744C

Last updated by author(s): Dec 29, 2020

Reporting Summary

Nature Research wishes to improve the reproducibility of the work that we publish. This form provides structure for consistency and transparency in reporting. For further information on Nature Research policies, see Authors & Referees and the Editorial Policy Checklist .

Statistics

For all statistical analyses, confirm that the following items are present in the figure legend, table legend, main text, or Methods section.

n/a	Confirmed	
☐	☒	The exact sample size (*n*) for each experimental group/condition, given as a discrete number and unit of measurement
☐	☒	A statement on whether measurements were taken from distinct samples or whether the same sample was measured repeatedly
☐	☒	The statistical test(s) used AND whether they are one- or two-sided<br>*Only common tests should be described solely by name; describe more complex techniques in the Methods section.*
☒	☐	A description of all covariates tested
☐	☒	A description of any assumptions or corrections, such as tests of normality and adjustment for multiple comparisons
☐	☒	A full description of the statistical parameters including central tendency (e.g. means) or other basic estimates (e.g. regression coefficient) AND variation (e.g. standard deviation) or associated estimates of uncertainty (e.g. confidence intervals)
☐	☒	For null hypothesis testing, the test statistic (e.g. *F*, *t*, *r*) with confidence intervals, effect sizes, degrees of freedom and *P* value noted<br>*Give P values as exact values whenever suitable.*
☒	☐	For Bayesian analysis, information on the choice of priors and Markov chain Monte Carlo settings
☒	☐	For hierarchical and complex designs, identification of the appropriate level for tests and full reporting of outcomes
☒	☐	Estimates of effect sizes (e.g. Cohen's *d*, Pearson's *r*), indicating how they were calculated

Our web collection on statistics for biologists contains articles on many of the points above.

Software and code

Policy information about availability of computer code

Data collection	Leica TCS SP5 LAS AF, qpcr, PLA assay, Axio-Imager Z1 Zeiss
Data analysis	Fiji, Matlab, IMARIS, R studio, Light Cycler 96, MetaSystems Isis

For manuscripts utilizing custom algorithms or software that are central to the research but not yet described in published literature, software must be made available to editors/reviewers. We strongly encourage code deposition in a community repository (e.g. GitHub). See the Nature Research guidelines for submitting code & software for further information.

Data

Policy information about availability of data

All manuscripts must include a data availability statement. This statement should provide the following information, where applicable:
- Accession codes, unique identifiers, or web links for publicly available datasets
- A list of figures that have associated raw data
- A description of any restrictions on data availability

Zenodo: doi 10.5281/zenodo.4415734

Field-specific reporting

Please select the one below that is the best fit for your research. If you are not sure, read the appropriate sections before making your selection.

☒ Life sciences ☐ Behavioural & social sciences ☐ Ecological, evolutionary & environmental sciences

For a reference copy of the document with all sections, see nature.com/documents/nr-reporting-summary-flat.pdf

Life sciences study design

All studies must disclose on these points even when the disclosure is negative.

Sample size	Sample sizes were determined either according to literature suggestions or based on the availability of lab resources and the respective statistical tests were chosen accordingly.
Data exclusions	No data were excluded from analyses.
Replication	The methods followed as described in the article allow the technical replication of all our experiments.
Randomization	Colocalization and protrusion size comparisons are observational studies. Confounding was not taken into account . In genomic and structural instability tests, fused AGO2-GFP expression vector was chosen randomly.
Blinding	Colocalization and protrusion size comparisons are observational studies. Confounding was not taken into account. In genomic and structural instability tests, AGO2 overexpression and knock down procedures, chromosomal measurements and statistical analyses were conducted by different researchers each blinded to preceding steps.

Reporting for specific materials, systems and methods

We require information from authors about some types of materials, experimental systems and methods used in many studies. Here, indicate whether each material, system or method listed is relevant to your study. If you are not sure if a list item applies to your research, read the appropriate section before selecting a response.

Materials & experimental systems

n/a	Involved in the study	
☐	☒	Antibodies
☐	☒	Eukaryotic cell lines
☒	☐	Palaeontology
☒	☐	Animals and other organisms
☒	☐	Human research participants
☒	☐	Clinical data

Methods

n/a	Involved in the study	
☒	☐	ChIP-seq
☒	☐	Flow cytometry
☒	☐	MRI-based neuroimaging

Antibodies

Antibodies used	Details provided in Table S2.
Validation	Data provided by the manufacturer and appropriate controls in Figure S7, S9.

Eukaryotic cell lines

Policy information about cell lines

Cell line source(s)	Cell lines were provided by ATCC and are stated in Supplemental Material.
Authentication	All the cell lines were authenticated by ATCC.
Mycoplasma contamination	All cell lines were mycoplasma free.
Commonly misidentified lines (See ICLAC register)	N/A
